# Single-cell transcriptome analysis of tumor and stromal compartments of pancreatic ductal adenocarcinoma primary tumors and metastatic lesions

**DOI:** 10.1186/s13073-020-00776-9

**Published:** 2020-09-29

**Authors:** Wei Lin, Pawan Noel, Erkut H. Borazanci, Jeeyun Lee, Albert Amini, In Woong Han, Jin Seok Heo, Gayle S. Jameson, Cory Fraser, Margaux Steinbach, Yanghee Woo, Yuman Fong, Derek Cridebring, Daniel D. Von Hoff, Joon Oh Park, Haiyong Han

**Affiliations:** 1 Molecular Medicine Division, Translational Genomics Research Institute, 445 N. Fifth St., Phoenix, AZ 85004 USA; 2 HonorHealth Research Institute, Scottsdale, AZ USA; 3 Samsung Medical Center, Sungkyunkwan University School of Medicine, Seoul, 06351 South Korea; 4 Department of Surgery, City of Hope National Medical Center, Duarte, CA USA

**Keywords:** Single-cell RNA sequencing, Pancreatic cancer, Cellular heterogeneity, Pancreatic cancer subtypes

## Abstract

**Background:**

Solid tumors such as pancreatic ductal adenocarcinoma (PDAC) comprise not just tumor cells but also a microenvironment with which the tumor cells constantly interact. Detailed characterization of the cellular composition of the tumor microenvironment is critical to the understanding of the disease and treatment of the patient. Single-cell transcriptomics has been used to study the cellular composition of different solid tumor types including PDAC. However, almost all of those studies used primary tumor tissues.

**Methods:**

In this study, we employed a single-cell RNA sequencing technology to profile the transcriptomes of individual cells from dissociated primary tumors or metastatic biopsies obtained from patients with PDAC. Unsupervised clustering analysis as well as a new supervised classification algorithm, SuperCT, was used to identify the different cell types within the tumor tissues. The expression signatures of the different cell types were then compared between primary tumors and metastatic biopsies. The expressions of the cell type-specific signature genes were also correlated with patient survival using public datasets.

**Results:**

Our single-cell RNA sequencing analysis revealed distinct cell types in primary and metastatic PDAC tissues including tumor cells, endothelial cells, cancer-associated fibroblasts (CAFs), and immune cells. The cancer cells showed high inter-patient heterogeneity, whereas the stromal cells were more homogenous across patients. Immune infiltration varies significantly from patient to patient with majority of the immune cells being macrophages and exhausted lymphocytes. We found that the tumor cellular composition was an important factor in defining the PDAC subtypes. Furthermore, the expression levels of cell type-specific markers for EMT^+^ cancer cells, activated CAFs, and endothelial cells significantly associated with patient survival.

**Conclusions:**

Taken together, our work identifies significant heterogeneity in cellular compositions of PDAC tumors and between primary tumors and metastatic lesions. Furthermore, the cellular composition was an important factor in defining PDAC subtypes and significantly correlated with patient outcome. These findings provide valuable insights on the PDAC microenvironment and could potentially inform the management of PDAC patients.

## Background

The tumor microenvironment (TME) which includes cellular and non-cellular components plays an important role in the progression, metastasis, and drug resistance of the tumors. The cellular components of TME (i.e., stromal cells) usually contain cells of hematopoietic origin (e.g., immune cells) and cells of mesenchymal origin (e.g., fibroblasts) [1]. The non-cellular components of TME include the extracellular matrix (ECM) and the signaling molecules produced by the cancer cells and stromal cells. Pancreatic ductal adenocarcinoma (PDAC), which accounts for > 90% of all pancreatic cancer cases, is one of the solid tumor types known to have a highly inflammatory and desmoplastic TME. The highly reactive and dense stroma contributes to the aggressiveness and drug resistance of PDAC, hence leading to the high mortality rate of the disease [2].

Recently, many drug development programs focus on developing stroma-remodeling agents for PDAC. Those agents either target the non-cellular components such as extracellular proteins (e.g., recombinant hyaluronidase that degrades hyaluronan) or aim to modulate the activity of certain stromal cell types such as cancer-associated fibroblasts and immune cells [3–6]. The rationale behind the development of these agents is that they will modulate the TME to a less fibrotic and/or less immunosuppressive state and thus lead to improved drug penetration and/or antitumor T cell infiltration. The accurate initial quantitative measurement of these components and the on-going effects of these stoma-targeted agents are critical to their successful development and therapeutic optimization.

Traditionally, measuring and phenotyping of cells in a solid tissue is done using immunohistochemical staining (IHC) and/or flow cytometry. Both techniques require high-quality antibodies against known cell type-specific marker proteins. Although IHC can provide spatial information on the cells within a tumor section, it is low throughput and not quantitative. FACS allows for single-cell analysis and is highly quantitative; however, it requires a large number of cells and, due to its reliance on fluorescent tags, issues including autofluorescence and spectral spillover can lead to loss of resolution. The recently developed cytometry by time-of-flight (CyTOF) has resolved the autofluorescence and spectral spillover issues and significantly improved the multiplexity [7, 8]. However, CyTOF is limited by the catalog of available isotope-labeled antibodies and generally also requires a high number of cells [9]. Recent advances in next-generation sequencing (NGS) technology have made the deep RNA sequencing at single-cell level feasible [10]. This allows the interrogation of whole transcriptome in individual cells within a tumor and the determination of their states at exceptionally high resolution. In this study, we employed a single-cell RNA sequencing (scRNA-Seq)-based profiling method to quantitatively determine the cell types and states within PDAC primary tumors and metastatic lesions to understand their heterogeneity and complexity.

## Methods

### PDAC tumor specimens

Fresh tumors from PDAC patients (Additional file 1: Table S1) were collected at HonorHealth Research Institute (Scottsdale, AZ, USA) and Samsung Medical Center (Seoul, South Korea) under Institutional Review Board-approved protocols. Signed informed consent was obtained from each patient. Primary tissues from ten different patients with localized PDAC were obtained during surgical resections, and the biopsies of six patients with metastatic PDAC (5 liver metastases and 1 omentum metastasis) were obtained by 19 gauge needles. Freshly harvested tissues were mechanically and enzymatically dissociated using a tumor dissociation kit (Cat #130-095-929, Miltenyi Biotec, Bergisch Gladbach, Germany) on a gentleMACS™ Dissociator (Miltenyi Biotec). After dissociation, single-cell suspensions were filtered using a 40-μm cell strainer to remove large pieces of debris. Red blood cells (RBC) were removed by incubating the cells with RBC lysis buffer (ThermoFisher Scientific, Carlsbad, CA). Cells were then counted and evaluated for viability using the trypan blue (0.4%) staining assay.

### Single-cell RNA sequencing (scRNA-Seq)

Single-cell whole transcriptome profiling of the dissociated tumor tissues was carried out using the Chromium Single Cell Gene Expression Solution system by 10x Genomics (Pleasanton, CA, US). Single cells were resuspended in PBS buffer at 10^6^ cells/mL and loaded onto the Chromium chips. The single-cell capturing, barcoding, and cDNA library preparation were performed using the Chromium Single Cell 3′ Library & Gel Bead Kit v2 by 10x Genomics by following protocols recommended by the manufacturer. The final sequencing libraries were checked for quality on Agilent 4200 Tapestation System and quantified by fluorometry staining (QuBit) assay. The libraries were sequenced on a HiSeq4000 (Illumina, San Diego, CA, USA) at a depth of ~ 50,000 reads per cell.

### scRNA-Seq data processing, quality control, and analysis

CellRanger (10x Genomics) was used to generate digital expression matrixes from the FASTQ files obtained from the Illumina sequencing runs. In addition to the filtering of low-quality barcodes (cells), we also removed cells with small library size (< 1000 UMI) or few expressed genes (Shannon diversity index < 3, calculated using the diversity function in the Vegan package in R) were excluded from further analysis. The digital expression matrices for cells that passed the quality control were then input into the Seurat R package (v3.0) [11, 12] to generate Seurat objects for the comprehensive downstream analyses and visualization. The following Seurat functions were used in the Seurat pre-processing pipeline: NormalizeData, ScaleData were used to calculate the comparable expression values; FindVariableFeatures were used to include the variable genes that contribute to the overall similarity/variability of cellular transcriptomic profiles; RunPCA, FindNeighbors, FindClusters, RunTSNE, and RunUMAP were used to calculate the dimension-reduction coordinates for visualization and to perform unsupervised clustering. In the downstream analyses, we used Uniform Manifold Approximation and Projection (UMAP) coordinates to visualize the layout of the cells.

### Modular score calculation and signal visualization

AddModuleScore function in the Seurat package was used to calculate the gene expression modular scores for each cell. Cells in the same cluster have a similar level of modular scores, indicating similar gene expression profiles and presumably similar cellular function or state. Cells were mapped onto dimension-reduction plots based on their modular scores using the FeaturePlot function in Seurat. Signal distribution in each cluster was evaluated and visualized using Violin plots using the VlnPlot function in Seurat.

### Cell type identification

To identify the cell types and subtypes that the tumors contain, two different methods were employed. The first method used the cell type markers that have been established in the literature [13, 14]. Those include EpCAM and KRT19 for ductal epithelial cells; COL1A1, ACTA2, and SPARC for fibroblasts; CDH2, SNAI2, and ZEB1 for EMT-like cells; CD3D, IL7R, and CD3G for lymphocytes; CD68 and G-CSF for monocytes/macrophages; KDR and VWF for endothelial cells; and FCER1A and CD1 for dendritic cells. The second was the supervised learning algorithm, SuperCT, which we described previously [15]. Briefly, a training set of 10x Genomics scRNA-seq data for ~ 200,000 cells representing 30 different cell types were used to train our SuperCT algorithm to establish the cell type prediction model. Once verified by additional datasets, the expression matrix files for the current study were input into the program. Each cell was assigned to one of the 30 different cell types (or unknown if does not match any of the 30 types) based on their expression profile. If the cells that were assigned to a particular cell type clustered together on the UMAP, then the cluster was identified as that cell type. This method allowed the identification of cell types that were not assigned using known cell markers.

Inferred copy number variation (CNV) analysis was carried out using the InferCNV R Package [16]. The stromal cancer-associated fibroblast cells were used as reference cells and the hidden Markov model was chosen to predict the CNV states.

### Gene Ontology term enrichment analysis

The hypergeometric test that is implemented in the R package “clusterProfiler” [17] was used to perform the enrichment analysis of differentially expressed genes or cell type-specific genes in Gene Ontology (GO) terms. The visualization function “dotplot” provided by clusterProfiler was used to generate the GO enrichment plots.

### Correlation analysis between cell type-specific gene signature and patient survival

Bulk RNA-seq datasets and the corresponding patient outcome (overall survival) data for PDAC patients were obtained from International Cancer Genome Consortium (ICGC) database (release 20 for US TCGA, Canada, and Australia cohorts) [18, 19]. To evaluate the correlation between the expression of cell type-specific signature genes and patient outcome, the top 20 signature genes (Additional file 2: Table S2) were first identified for each cell type (cluster) using the FindMarkers function in the Seurat package (FindMarkers ranks the genes based non-parametric Wilcoxon rank sum tests between the cell type of interest and the rest of the cell types). An expression matrix was then generated for each of the 20 genes across the patients based on their expression levels. For a given patient, if the expression level of a given gene was equal to or greater than the median expression of the gene across all patients, then the matrix value for that gene in that patient was assigned as 1. If the expression level was less than the median expression value, then the matrix value was assigned as 0. For each patient, the matrix values for the 20 signature genes were then added up to obtain an overall expression score for the gene signature (and thus the cell type). The patients were then classified as high and low for the specific cell types, if their overall expression score fell into the top 25% quartile and the low 25% quartiles, respectively. The overall survival of the patients in those two high and low expression groups was finally compared using the Kaplan–Meier curves and log-rank *P* value analysis.

### SuperCT cell type classification

SuperCT is a cell type classifier we previously described [15]. It utilizes a machine-learning algorithm to establish a cell type classifier from the published single-cell RNA-seq datasets and then uses the classifier to predict the cell types in the new single-cell RNA-seq datasets that have a similar biological context. The cell-classifier is independent of the dimension-reduction and unsupervised clustering methods, which allows the identification of small cell populations that do not form distinct clusters. In this study, we used the v1i version of SuperCT to characterize the cell types for the total single-cell RNA-seq datasets [15]. To further characterize the T cell populations, we used the single-cell RNA-seq dataset of melanoma-associated T cells reported by Li and coworkers to train our SuperCT immune cell module and then used it to predict the T cell subtypes in the PDAC tumor tissues [20].

### Statistical analysis

The Kaplan–Meier survival curves were plotted using GraphPad Prism 8. The log-rank (Mantel–Cox) test was used to determine the *P* value (< 0.05 is considered to be significant), hazardous ratio (HR), and 95% confidence interval (CI).

## Results

### scRNA-Seq profiling reveals heterogeneous cell composition in PDAC tissues

We obtained fresh primary tumor tissues from 10 patients with resected PDAC and core needle biopsies from the metastatic lesions (liver or omentum) of 6 patients with metastatic PDAC. The tumors were histologically confirmed as PDAC. The clinicopathological characteristics of the patients are summarized in Additional file 1: Table S1.

The tissues were processed and dissociated into single-cell suspension and sequenced using the Chromium single-cell RNA-Seq platform (10x Genomics). After stringent quality control and normalization analysis, we obtained high-quality transcriptomic profile data from a total of 8000 cells from the 10 primary tumors and 6926 cells from the 6 metastasis samples. The number of cells obtained from each patient ranges from 143 to 1570 for primary tumors and 125 to 2885 for metastatic biopsies.

**Fig. 1:**
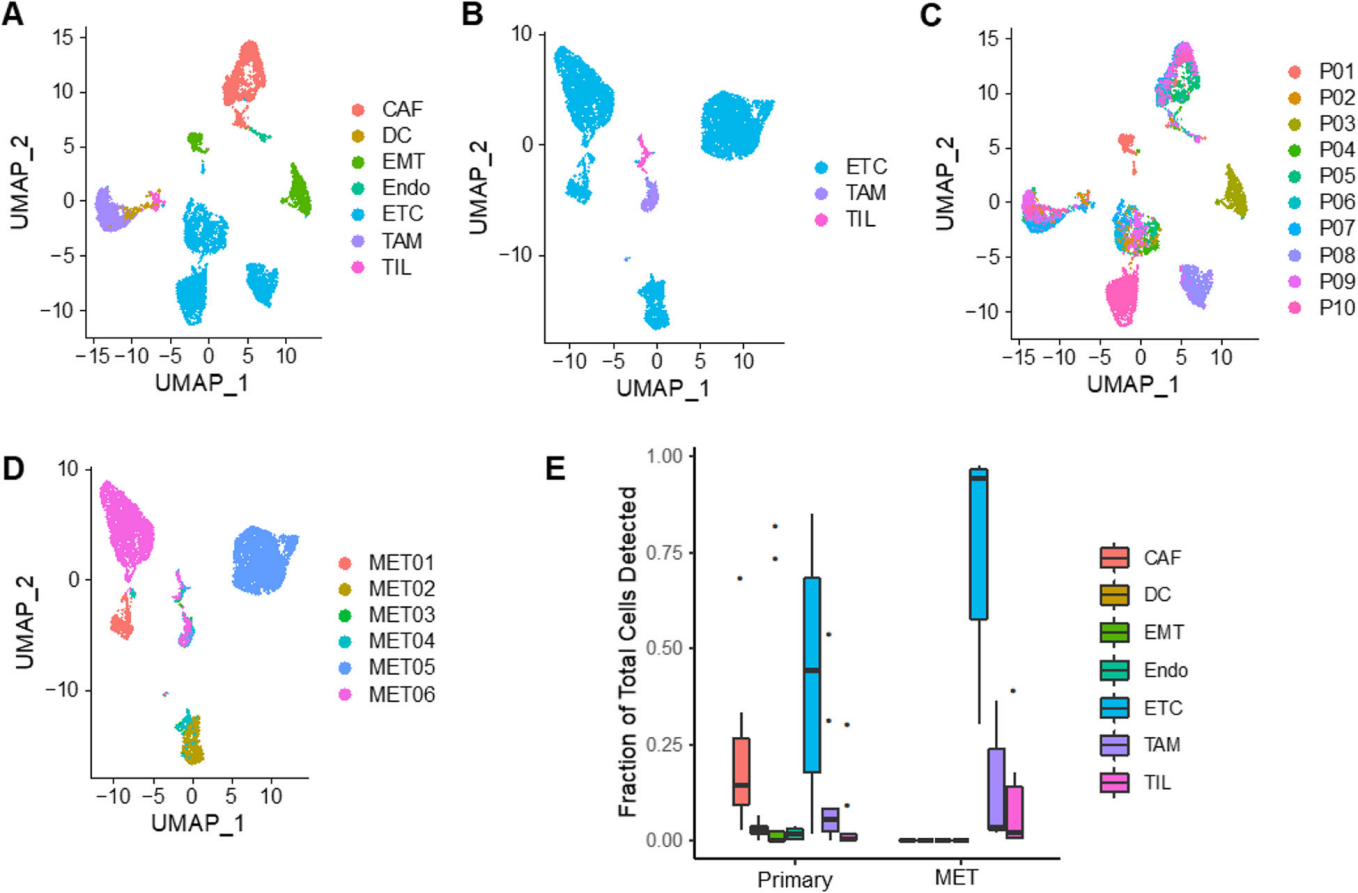
Multiple cell types were identified in PDAC primary tumors and metastatic lesions by single-cell RNA sequencing (scRNA-Seq). The cells from PDAC primary tumors (**a**, **c**) or metastatic lesions (**b**, **d**) were analyzed using unsupervised clustering and visualized using a UMAP plot. The clusters in **a** and **b** are color-coded based on cell types identified using known cell type-specific markers. The clusters in **c** and **d** are color-coded based on the patients. **e** A box plot showing the distribution of each cell type in the primary tumors and metastatic biopsies (MET)

Using unsupervised clustering analysis and the Uniform Manifold Approximation and Projection (UMAP), a dimension-reduction and visualization method [21], we were able to identify segregated cell clusters in the primary PDAC tissues. To identify the cell identities that those clusters represent, we used known cell type markers previously established to classify the major clusters or the signature-enriched populations into different cell types including epithelial cells, fibroblasts, endothelial cells, and immune cells (Fig. 1).

The epithelial cells express epithelial cell adhesion molecule (EpCAM) and cytokeratin 19 (KRT19). To verify that those cells are indeed epithelial tumor cells, we determined their cell cycle status using validated genes previously shown to identify cells in active cell cycling phases (G1/S and G2/M) [22] and found that a much higher fraction of those cells was in an active cell cycling process in comparison to the normal epithelial cells and the fibroblast cells. The fraction of the epithelial cells in active cell cycling is also comparable to that reported for pancreatic ductal tumor cells by Peng et al. [13], further supporting the assignment of those epithelial cells as tumor cells (Additional file 1: Fig. S2). Therefore, we designated those cells as epithelial tumor cells (ETCs). The fibroblast cells express the well-known fibroblast-related genes such as collagens (COL1A1, COL1A4), SPARC, and alpha smooth muscle Actin (ACTA2). We designate those fibroblasts as cancer-associated fibroblasts (CAFs). We identified 3 major clusters of immune cells that include BDCA-1^+^ dendritic cells (DC), CD14^+^/CD68^+^ macrophages, and CD3^+^ T cells. We designated the macrophages as tumor-associated macrophages (TAMs) and the T cells as tumor-infiltrating lymphocytes (TILs). The endothelial cells (Endo) represented a minor cell population that expressed CD34, VWF, and KDR genes. Interestingly, a subset of clusters showed a strong epithelial to mesenchymal transition (EMT) signal (Additional file 1: Fig. S3). This cluster also demonstrated a high proliferative signal (Additional file 1: Fig. S2). We therefore designated them as tumor cells with EMT characteristics (EMT, Fig. 1). To further verify the assignment of the cell types, we performed inferred gene copy number analysis using the InferCNV software [16]. As can be seen in Additional file 1: Fig. S4, when using CAF cells as the reference, the copy number profiles inferred from the single-cell RNA sequencing are very similar between the CAFs and the different subsets of immune cells, whereas the cancer cells (both the epithelial tumor cells and the tumor cells have undergone EMT) showed substantial copy number variations (Fig. S4). This result is consistent with the fact that pancreatic cancer cells are highly aneuploid with numerous copy number alterations and the stromal cells are generally diploid with few copy number variations.

Overall, the human primary PDAC tumors contained 7 major cell populations including ETCs, EMTs, CAFs, DCs, Endos, TILs, and TAMs (Fig. 1a). The metastatic lesions contained 3 major cell populations including ETCs, TILs, and TAMs. It is noteworthy that the tumor cells (ETCs and EMTs) from different patients tend to form separate clusters in both primary tumors and metastatic lesions, suggesting that tumor cells from different patients have significant heterogeneity (Fig. 1c, d). The CAFs and immune cells from different patients are mostly mixed together. As a quality measurement, we also evaluated the distribution of the fraction of mitochondrial genes in each cell type. As shown in Additional file 1: Fig. S5, the fraction of mitochondrial genes is very low (< 2.0% of the total genes) with the TILs having a relatively highly percentage than the other cell types.

The fraction of each cell type in a given patient varied greatly from patient to patient (Table 1 and Fig. 1e), which adds another level of heterogeneity among patients. In human primary tumors, the percentage of ETCs ranges from less than 1.7 to 85.1% (average 42.2%), the CAFs from 2.8 to 68.3% (average 21.7%), TAMs from 0.5 to 53.7% (average 11.84%), and TILs from 0 to 30.1% (average 4.3%). The metastases contained a higher percentage of epithelial tumor cells (30.4–97.6%) with varying percentage of immune cells (TAMs and TILs). It is worth noting that patients with a higher number of TILs also have a higher number of TAMs (Table 1).

**Table 1:** Cell types and abundancies in PDAC primary tumors and metastatic lesions detected by scRNA-Seq

		Percentage of total cells detected						
Patient ID	Primary/metastasis	CAF	DC	EMT	Endo	ETC	TAM	TIL
P01	Primary	8.6	3.7	73.1	3.2	9.3	0.5	1.2
P02	Primary	2.8	6.3	0.7	0.0	51.7	8.4	30.1
P03	Primary	10.7	0.1	82.0	3.7	1.7	1.6	0.1
P04	Primary	24.6	< 0.1	2.8	< 0.1	72.7	< 0.1	< 0.1
P05	Primary	68.3	1.8	< 0.1	2.3	17.7	8.1	1.8
P06	Primary	33.4	3.8	< 0.1	3.1	55.1	4.2	0.4
P07	Primary	14.1	4.5	< 0.1	1.0	17.6	53.7	9.1
P08	Primary	14.3	3.4	< 0.1	1.2	74.0	6.7	0.4
P09	Primary	27.2	1.8	< 0.1	2.8	37.0	31.1	0.2
P10	Primary	8.7	2.0	< 0.1	< 0.1	85.1	4.1	0.1
MET01	Liver Met	< 0.1	< 0.1	< 0.1	< 0.1	97.6	2.1	0.2
MET02	Liver Met	< 0.1	< 0.1	< 0.1	< 0.1	97.0	2.7	0.3
MET03	Omentum Met	< 0.1	< 0.1	< 0.1	< 0.1	30.4	30.4	39.2
MET04	Liver Met	< 0.1	< 0.1	< 0.1	< 0.1	45.8	36.5	17.7
MET05	Liver Met	< 0.1	< 0.1	< 0.1	< 0.1	95.6	3.2	1.2
MET06	Liver Met	< 0.1	< 0.1	< 0.1	< 0.1	93.4	3.8	2.8

*Met* metastasis, *CAF* cancer-associated fibroblast, *DC* dendritic cell, *EMT* epithelial to mesenchymal transition tumor cell, *Endo* endothelial cells, *ETC* epithelial tumor cell, *TAM* tumor-associated macrophage, *TIL* tumor-infiltrating lymphocyte

To identify minor cell types that do not form distinct clusters, we also used a supervised classification method, SuperCT [15]. This approach allowed more robust identification of cell types with small cell numbers and subtypes of major cell types (Additional file 1: Fig. S1). A small population of acinar cells was identified. Multiple additional subtypes of immune cells such as B cells and natural killer (NK) cells were also identified, even though there are no obvious cluster segregations for those cell types in the UMAP or tSNE plots. Interestingly, the SuperCT algorithm assigned a significant number of epithelial tumor cells (EpCAM^+^ and KRT19^+^) as an unknown cell type. This is due to the fact that the SuperCT v1i was trained on a dataset that only included normal tissues and the majority of epithelial tumor cells apparently did not resemble the normal epithelial cells or other cell types defined in the classifiers. This observation demonstrates the high robustness of the SuperCT tool.

### Epithelial tumor cells in PDAC exhibit a high inter-patient heterogeneity

**Fig. 2:**
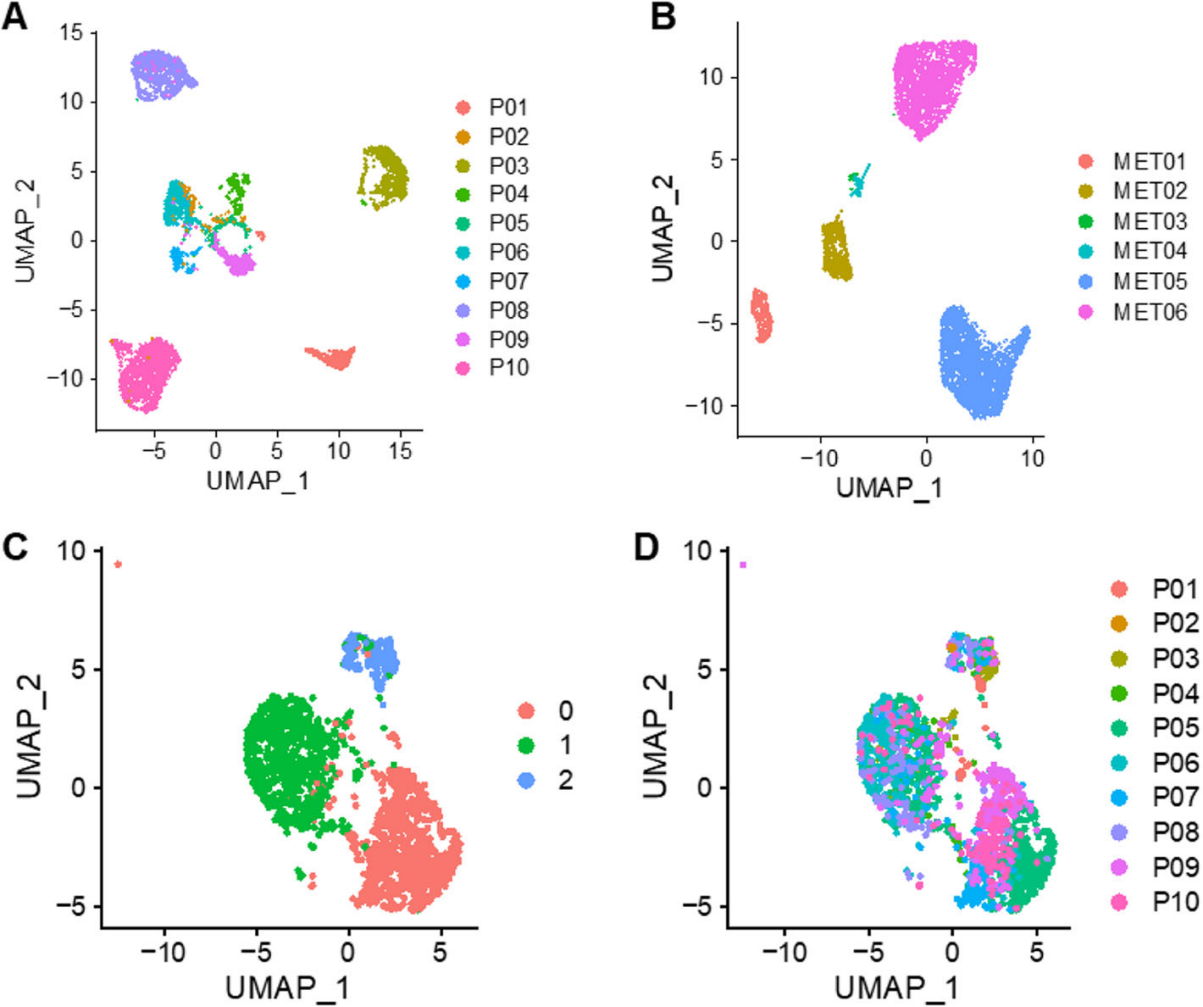
Unsupervised clustering analysis of tumor cells and cancer-associated fibroblasts (CAFs) in PDAC primary tumors and metastatic lesions. **a** Tumor cells in the primary tumors are mostly segregated by patients. **b** Tumor cells in the metastatic lesions also cluster by patients. **c** Three major clusters are formed by CAFs from primary tumors. **d** CAFs from different patients are mixed in the different clusters

To further characterize the tumor cells, we pooled the tumor cells from all primary tumors and clustered them unsupervised. As shown in Fig. 2a, the tumor cells clustered together primarily by patient, indicating significant inter-patient heterogeneity. Similar clustering patterns have been seen in single-cell sequencing analysis of other solid tumor types such as triple-negative breast, melanoma, and glioblastoma [16, 22, 23]. Two of the clusters have high expression of genes related to mesenchymal phenotype (e.g., CDH2, SNAI2, ZEB1, TWIST1, and VIM) which were assigned as tumor cells with EMT characteristics (EMT^+^). The two EMT clusters were traced back to 2 different patients and well separated from each other. Some of the tumor cells expressed stem cell markers (e.g., PROM1) but did not form separate clusters (Additional file 1: Fig. S6). The EMT cluster looks to be more closely associated with CAFs than the ETCs on the UMAP. We therefore performed pathway enrichment analysis to further characterize their differences. The unique signature genes (Additional file 3: Table S3) that define the CAF and EMT cell populations were input into the Ingenuity Pathway Analysis (IPA) core analysis tool (Qiagen, Inc., Germantown, MD). As shown in Additional file 1: Fig. S7, the top ten canonical pathways enriched for the signature genes are very different between the two cell populations. The CAF signature genes are more enriched in pathways related to extracellular matrix and fibrosis, whereas the EMT signature genes are more enriched in pathways related to cancer signaling and cell cycle.

Similar to those from primary tumors, tumor cells from metastatic biopsies also segregated by patient in unsupervised clustering analysis (Fig. 2b). Notably, the tumor cells from the metastatic lesions showed little mesenchymal characteristics (Additional file 1: Fig. S8). Interestingly, one of the tumors showed strong signal characterized of acinar cells based on the SuperCT model prediction (Additional file 1: Fig. S9A) and are highly positive for expression of acinar cell maker genes (Additional file 1: Fig. S9B-C).

When the cells from the primary tumors and metastatic lesions were combined and clustered unsupervised, the segregation pattern of the different cell types stayed the same. The epithelial tumor cells from different patients remained segregated (Additional file 1: Fig. S10). The stromal cells were generally clustered together based on cell types (TAMs, TILs, CAFs, etc.) regardless of their patient of origin or tissue type (primary or metastatic).

### Cancer-associated fibroblasts support an inflammatory and fibrotic tumor microenvironment in PDAC

To further investigate the biology of CAFs, we pooled the CAF cells from the human PDAC primary tumors for analysis. Figure 2c shows the unsupervised clustering of 1753 CAF cells identified in Fig. 1a. The CAFs formed 3 major clusters: c0, c1, and c2 (Fig. 2c). Unlike the tumor cells, the CAFs did not cluster by patient. Each cluster contained CAFs from different patients (Fig. 2d). This finding indicates that CAFs from different patients were more similar in their gene expression profiles than their companioning tumor cells. The CAFs expressed high levels of collagen genes, SPARC, and α-smooth muscle action (ACTA). Elyada and colleagues previously described 3 subtypes of CAFs identified in PDAC tumors: myofibroblasts (myCAFs), inflammatory fibroblasts (iCAFs), and antigen-presenting fibroblasts (apCAFs) [24]. We therefore set out to determine whether or not any of CAF clusters identified in our analysis belong to those 3 subtypes. Cluster 0 expresses POSTN and MMP11, which were reported to be expressed mainly in myCAFs [25]. However, Clusters 1 and 2 do not seem to enrich the signature genes associated with iCAF or apCAFs. The signature genes that define cluster 1 are more enriched with genes associated with quiescent (or normal) CAFs. Interestingly, cluster 2 displays an expression signature that resembles smooth muscle cells (enriched for RGS5, NOTCH3, and CSRP2 expression) based on the SuperCT analysis (Additional file 1: Fig. S11). These cells might be the mural cells including pericytes and vascular smooth muscle cells from the blood vessels [26].

### Immune suppressive cells in PDAC maintain a tumor-friendly environment

**Fig. 3:**
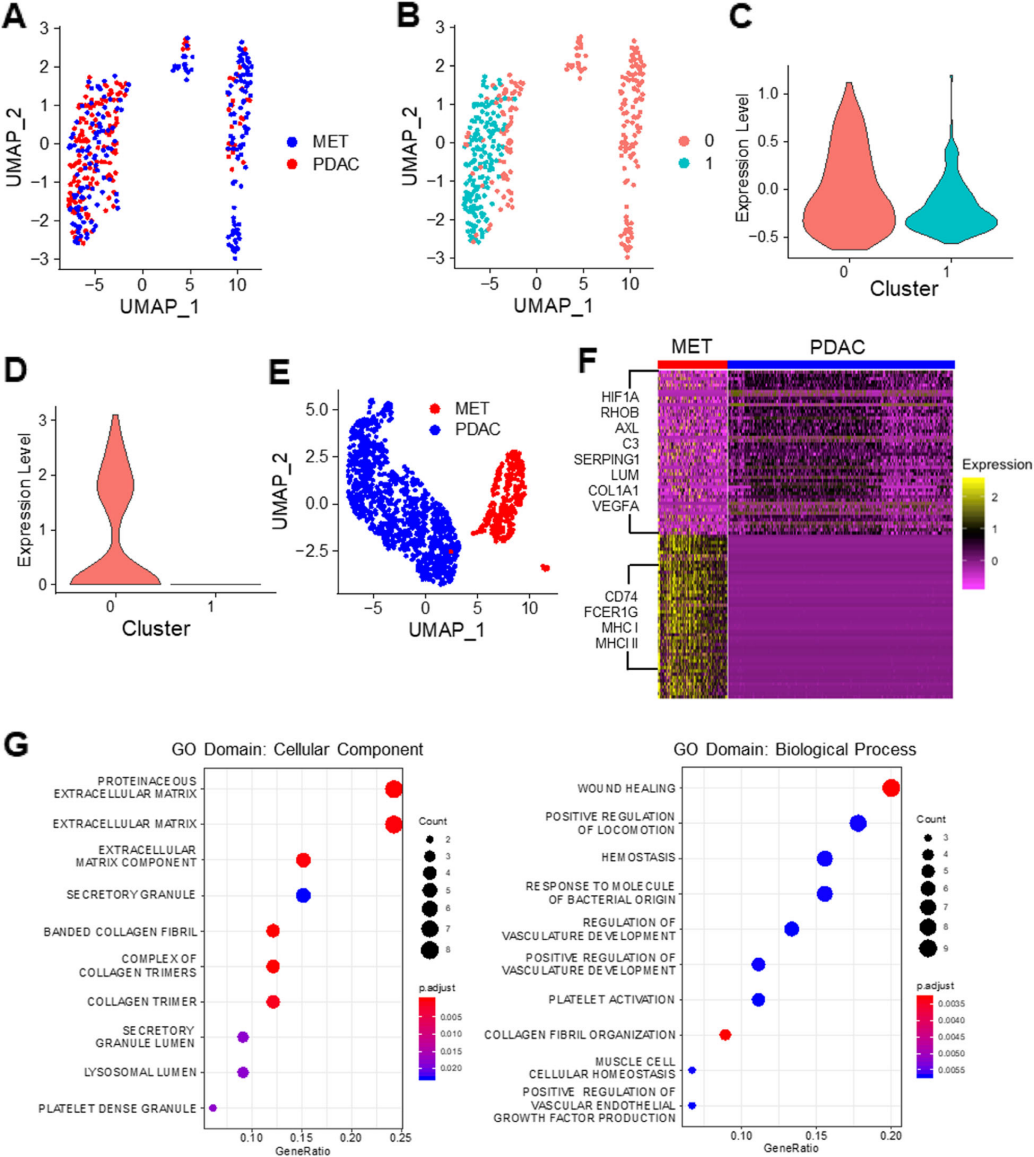
Unsupervised clustering analysis of immune cells in PDAC primary tumors and metastatic lesions. Tumor-infiltrating lymphocytes (TILs) from primary tumors and metastatic lesions are mixed together (**a**) and form two main clusters (**b**). One of the clusters (c0) showed higher expression of genes associated with T cell exhaustion (**c**) and those cells also express a higher level of Ki67 gene (**d**). The tumor-associated macrophages (TAMs) from primary tumors and metastatic lesions form separate clusters (**e**). Heatmap shows distinct gene expression patterns between the two TAM populations (**f**) and the genes specifically express in the TAMs associated with the primary tumors are enriched in processes related to extracellular matrix (left panel in **g**) and wound healing (right panel in **g**). The expression level (*Y*-axis) in **c** and **d** is the logarithm-transformed ratio of the UMI counts of the gene(s) of interest over the total UMI counts in each individual cell. GO Gene Ontology

Three major immune cell types were identified in the human primary tumors and metastatic biopsies: lymphocytes (TILs), macrophages (TAMs), and dendritic cells (DCs) (Fig. 1a). To examine the functional activity of the lymphocytes, we extracted the TILs and clustered the cells using unsupervised clustering. As shown in Fig. 3a, the TILs from primary tumors and metastases were mixed together, indicating their similar functional states and phenotypes. Only a few of TILs were CD8^+^ (Additional file 1: Fig. S12A). However, TILs were separated into two clusters (Fig. 3b). One of the clusters (c0) showed higher levels of expression of exhaustion markers such as TIGIT, CTLA4, PDCD1, HAVCR2, LAG3, and LAYN (Fig. 3c), indicating that those cells were exhausted with limited effector function. However, those T cells were still proliferative as they were highly Ki67 positive compared to the other T cell clusters (cluster 1, Fig. 3d). We also utilized the SuperCT framework to establish a predictive model for TIL subtypes based on a scRNA-Seq dataset obtained from melanoma tissues [20]. Using this SuperCT model, several TIL subtypes including predictions provided more details on the TIL subpopulations. (Additional file 1: Fig. 12B). The c1 cluster seemed to be enriched with the naïve-like T cell subtype (Additional file 1: Fig. S12C).

In contrast to the TILs in which the cells from the primary tumors and metastases were mixed, macrophages from the two different sample types clustered separately, suggesting that the macrophages in the primary tumors and in the metastasis are very different (Fig. 3e). The macrophages from the primary tumors showed high expression of HIF1A, RHOB, AXL, C3, SERPING1, LUM, COL1A1, and VEGFA (Fig. 3f). Gene set enrichment analysis showed that those genes were enriched in extracellular matrix and late stages of the wound healing-related processes, which is characteristic of M2-like macrophages (Fig. 3g) [27]. The macrophages in the metastases, on the other hand, expressed genes such as CD74, FCER1G, and MHC I/II-related genes that are related to the antigen-presenting function of macrophages (Fig. 3f) [28]. Since besides hematopoietic stem cells TAMs can also be derived from tissue-resident macrophages [29], the difference between the TAMs from liver metastatic lesions and the primary tumors observed in our study could be, in part, due to the intrinsic differences between liver-resident macrophages and pancreas-resident macrophages. Further studies are needed to verify this possibility.

### Cell population composition might dictate PDAC subtype

Several recent studies have described the distinct PDAC subtypes based on transcriptomic profiling of bulk tissues. Collisson and coworkers defined three subtypes: classic, quasi-mesenchymal (QM), and exocrine; Moffitt and colleagues identified two subtypes: basal and classic; and Bailey et al identified four subtypes: squamous, pancreatic progenitor, immunogenic, and aberrantly differentiated endocrine exocrine (ADEX) [30–32]. To investigate whether the gene expression signatures from the bulk tissue analysis that define the subtypes are enriched in certain cell types within the tumor, we used modular scores (see the “Methods” section for details) to evaluate the overall expression of signature genes that define each subtype in those studies in each cell type identified in our scRNA-Seq analysis of the 10 primary PDAC tumors.

**Fig. 4:**
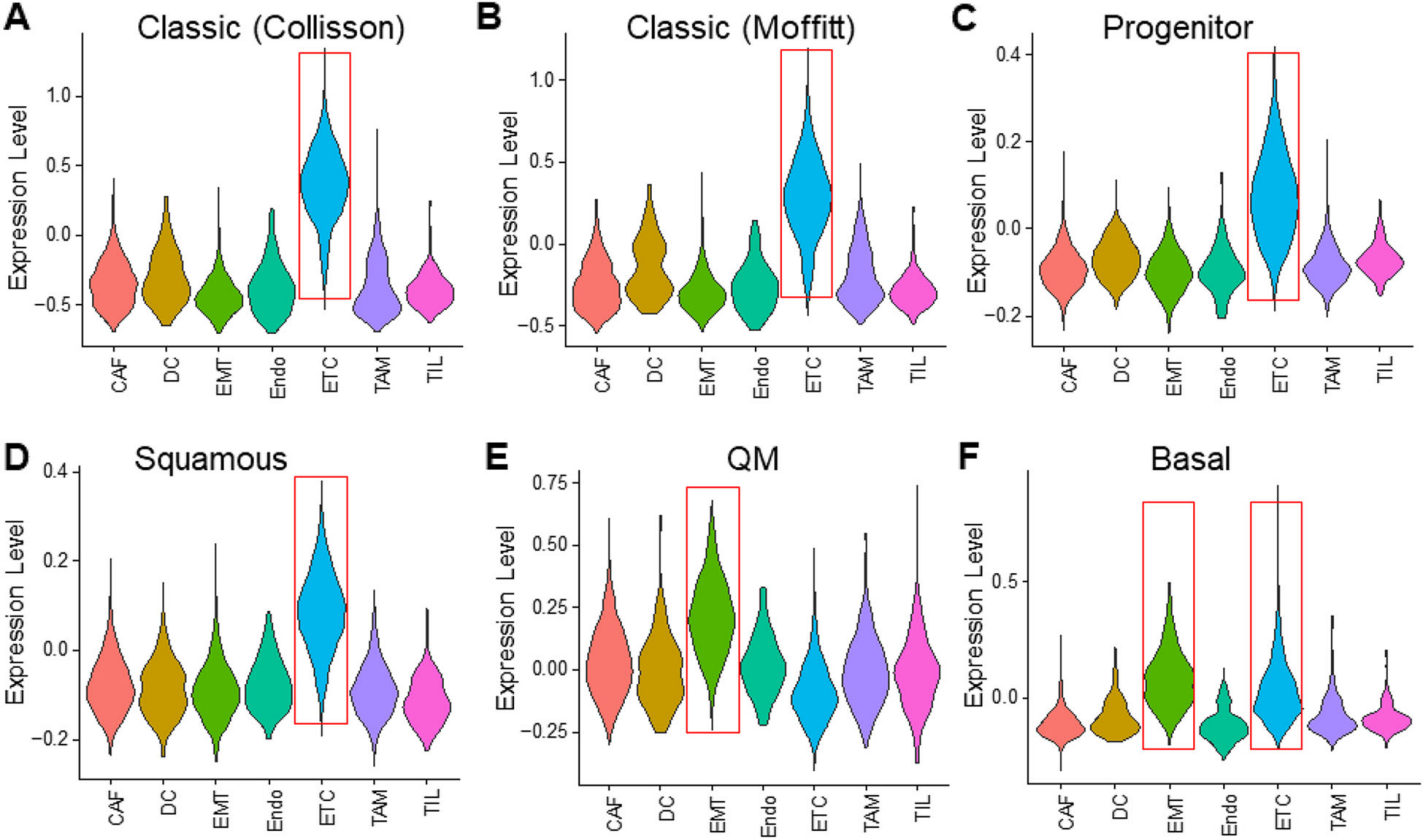
Expression of PDAC subtype signature genes in different cell types identified by single-cell transcriptomics. Violin plots are used to show the modular expression scores of the signature genes that define subtypes described previously: the classic subtype described by Collisson et al. (**a**) and Moffitt et al. (**b**), the progenitor (**c**) and the squamous subtypes by Bailey et al. (**d**), the QM subtype by Collisson et al. (**e**), and the basal subtype by Moffitt et al. (**f**). Red boxes indicate cell types that have higher expression scores than the other cell types

Figure 4 shows the violin plots of the modular scores of genes defining the different subtypes in the 7 major cell types identified in our scRNA-Seq analysis. It is apparent that the genes defining the classic subtype in the Collisson and Moffitt studies and the progenitor and squamous subtypes in the Bailey study were enriched in the epithelial tumor cell (ETC) population (Fig. 4a–d). The QM subtype signature genes were highly enriched in the EMT tumor cells, whereas the basal subtype genes defined in the Moffitt study were enriched in both ETC and EMT tumor cells (Fig. 4e, f). The signature genes defining the immunogenic subtype seemed to enrich in the dendritic cells and TILs (Additional file 1: Fig. S13). However, this initial analysis of the 7 major cell types did not identify cell types that were enriched for the exocrine and ADEX subtypes. We therefore examined the additional cell types identified in our SuperCT analysis. The signature genes for both the exocrine and ADEX subtypes were highly enriched in the acinar cells (Additional file 1: Fig. S14), indicating that the gene expression signals for these two subtypes might have come from the acinar cells in the bulk tumor tissues.

Interestingly, within individual patients, the overall expression level for the classic, progenitor, and squamous subtypes described by Collisson and Bailey, respectively [30, 31], was similar for all three profiles (e.g., relatively high expression levels in Patients P02, P06, P08, and P10), further suggesting the similarity of gene signatures among those subtypes (Additional file 1: Fig. S15). Patients P01, P04, P06, P08, P09, and P10 showed a high number of cells that express those signature genes, which is consistent with the fact that those patients have high number of ETC cells. Similarly, the number of cells expressing QM subtype signature was high in patients 1 and 3 whose tumor have high number of EMT tumor cells (Additional file 1: Fig. S16A). Finally, 2 (patients 2 and 7) out of the 3 patients (patients 2, 7, and 9) whose tumors had a relatively large number of immune cells (Table 1) show high levels of expression of Immunogenic subtype signature genes (Additional file 1: Fig. S16B). These results indicate that when using expression data from bulk tumor tissues, the composition of different cell types within the tumor might be a determining fact in defining the tumor subtypes.

To further examine the relationship between cell type composition and subtype classification, we used the expression levels of 144 signature genes that define the different PDAC molecular subtypes reported in Collisson et al. [30], Bailey et al. [31], and Moffitt et al. [32] to perform unsupervised clustering analysis of the cells from the primary tumors. As expected the signature genes were able to clearly identify the epithelial tumor cells and EMT cells (Additional file 1: Fig. S17A). More interestingly, the signature genes were also able to separate the CAFs and immune cells from each other and from the cancer cells. When viewed by patients, the cancer cells from the two patients with high number of EMT cells were well separated from ETCs from the rest of the patients (Additional file 1: Fig. S17B). The immune cells and CAFs from different patients are generally mixed and did not form subclusters, indicating that the signature genes were not able to differentiate the subsets within these two cell types. These results suggest that some of signature genes are expressed in immune cells or CAFs; therefore, their expression levels in bulk tissue transcriptome analysis could be influenced by tumor stromal content.

### Tumor cellular composition is associated with patient survival

**Fig. 5:**
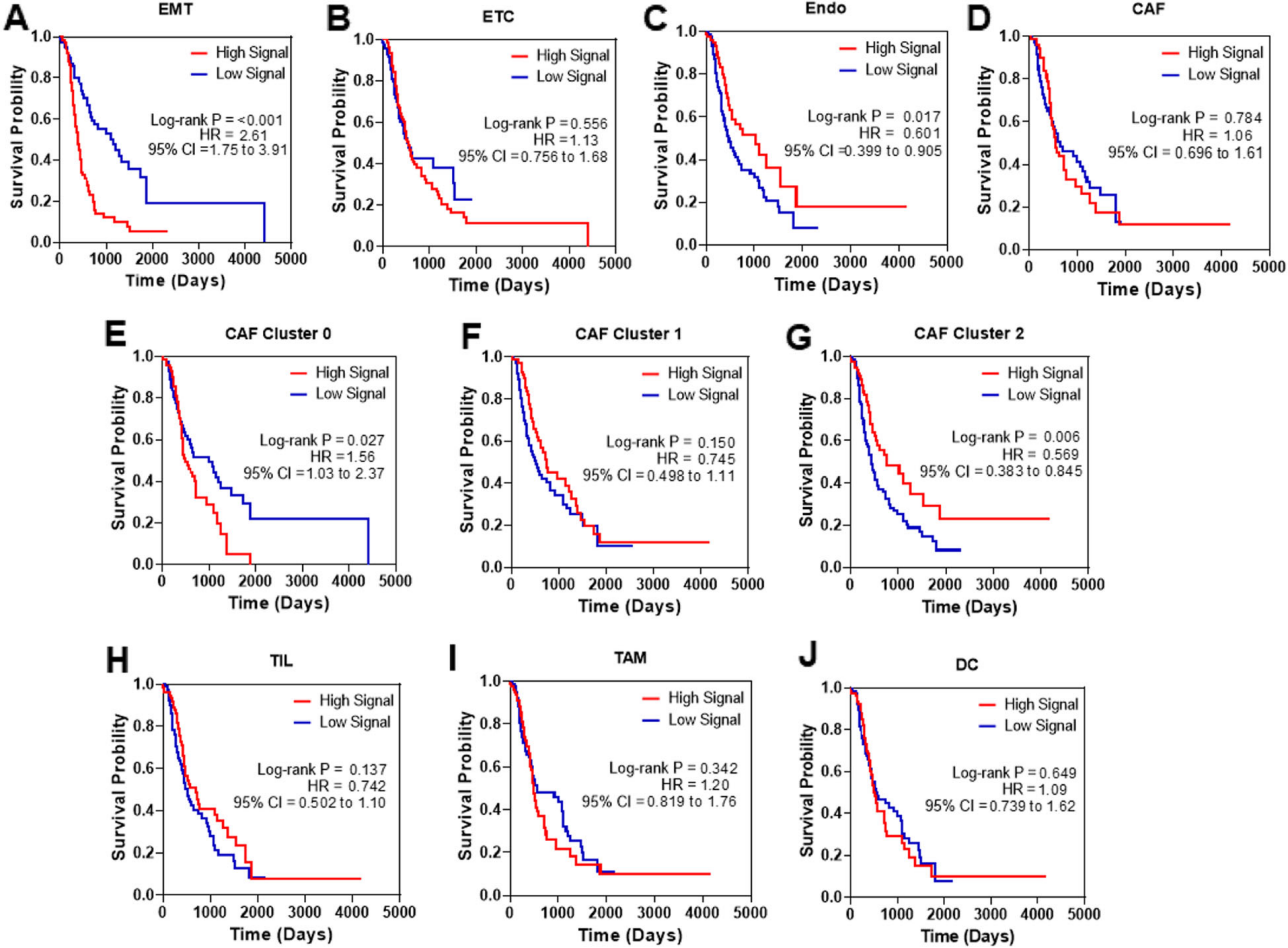
Kaplan–Meier survival curves for PDAC patients in the ICGC database by expression levels of cell type-specific gene signatures derived from the single-cell transcriptomics analysis. **a** EMT cell gene signature. **b** ETC cell gene signature. **c** Endothelial cell gene signature. **d** CAF gene signature. **e** CAF cluster 0 gene signature. **f** CAF cluster 1 gene signature. **g** CAF cluster 2 gene signature. **h** TIL gene signature. **i** TAM gene signature. **j** Dendritic cell gene signature

To further test the hypothesis that the composition of cell populations within a tumor is indicative of tumor progression and disease state, we next derived gene signatures that defined each of the cell types identified in the primary tumors based on the scRNA-Seq analysis (Additional file 2: Table S2). These gene signatures were then applied to publicly available bulk RNA-seq profiles from PDAC patients whose survival outcomes are available (the US TCGA, Canadian, and Australian cohorts in the ICGC database). The expression levels (scores) of the cell type-specific gene signatures were calculated from the bulk RNA-seq data for each patient (see the “Methods” section). When the expression scores of the cell-specific signature genes were correlated with patient survival, we observed statistically significant associations between the gene expression and overall patient survival for certain cell types. Firstly, high expression of EMT tumor cell signature genes significantly associated with shorter patient survival [*P* value < 0.0001, hazardous ratio (HR) = 2.76, Fig. 5a]. This is not surprising as EMT is generally considered to be a process that leads to more aggressive and invasive disease and tumor cells undergone EMT are associated with drug resistance [33, 34]. However, the ETC gene signature was not associated with patient survival (Fig. 5b). These findings are consistent with what was reported by Collisson et al. that the QM subtype of PDAC (corresponding to patients with high EMT cell population), but not the classic subtype (corresponding to patients with high ETC cell population), had shortened patient survival [30]. Secondly, high endothelial cell signature is significantly associated with better patient survival (*P* value = 0.017, HR = 0.6, Fig. 5c). This observation supports the notion that PDAC is generally hypovascularized and that improved vascularization could lead to higher drug perfusion and thus higher treatment efficacy and patient survival. Thirdly, the correlation between the expression of gene signature specific for the total CAFs and patient survival was not statistically significant (Fig. 5d). However, when we examined the gene signatures that were specific to the CAF subclusters, we found that high expression of the signature genes for cluster 0 which were the activated CAFs (Additional file 1: Fig. S18) was significantly associated with poorer patient survival (*P* value = 0.027; HR = 1.56, Fig. 5e). The gene signature for the other major CAF cluster, cluster 1, was not significantly associated with patient survival (Fig. 5f). Finally, the expression of the gene signatures for the TAMs, TILs, or DC immune cells was not significantly associated with patient survival (Fig. 5g, h). Collectively, these findings indicate that different cell types within tumor display distinct biology and their abundance can confer favorable or poor clinical outcomes in patients with pancreatic cancer.

## Discussion

In this study, we identified 7 predominant cell populations in the primary tumor tissues that included 2 tumor cell populations, 3 immune cell populations, endothelial cells, and fibroblasts, and 3 major cell populations in the metastatic biopsies that included one tumor cell population and 2 immune cell populations through single-cell transcriptome analysis on patient-derived primary and metastatic PDAC tumors. Although the expression profiles of the tumor cells in primary tumors and metastases were very different, the immune cells (T cells and macrophages) from different patients in those two tissue types showed high similarity. Previous studies have used scRNA-Seq to characterize the cell populations in human and mouse primary PDAC tumors [13, 24, 35]. Although those studies have identified the similar major cell types (e.g., tumor cells, fibroblasts, and immune cells) and the heterogeneous nature of PDAC primary tumors, our study revealed more detailed information on each of the cell types with the identification of additional cell subtypes. To our knowledge, this study is the first to perform single-cell transcriptome analysis on fresh biopsies from PDAC metastasis. We revealed that the cellular landscape of PDAC metastases might not be as complex as the primary tumors with the identification of only 3 predominant cell populations. Interestingly, very few cancer-associated fibroblasts were identified in the metastatic tumor tissues. Although these observed cellular compositions could have been resulted from the sampling bias of core needle biopsy, the fact that they contain a significant number of immune cells indicates that these findings might have biological significance and could potentially have implications in the selection of treatment strategies for patients with metastatic disease.

We found that the tumor cells in both primary tumors and metastases clustered based on their patient of origin (Fig. 2). One plausible explanation for this segregation pattern of the tumor cells is the potential batch effects in the tissue dissociation and single-cell RNA sequencing process. However, the fact that other cell types (fibroblasts, endothelial cells, and immune cells) did not display such patient-based segregation patterns indicates that this was not a result of technical artifacts. In fact, similar patterns have been observed in other tumor types including breast, melanoma, and glioblastoma [16, 22, 23]. Despite their inter-patient differences in gene expression patterns, the tumor cells can be classified into two subpopulations: one with epithelial characteristics and the other with EMT characteristics (Fig. 1c, d). The existence of EMT^+^ tumor cell population seems to be associated with more aggressive disease and poorer patient prognosis (Fig. 5). This observation is consistent with a recent study in which authors identified two tumor cell populations in murine PDAC primary tumors: one enriched for epithelial markers and the other enriched for mesenchymal markers with the later population mainly existing in advanced PDAC [35]. We did not identify a significant number of tumor cells with mesenchymal characteristics in the metastatic tumor specimens. This observation is consistent with the two-step metastasis model in which tumor cells undergo EMT first to gain the ability to migrate to and invade surrounding tissues and travel through the circulation. Once arrived at the distant organ, the tumor cells undergo a process termed mesenchymal-epithelial transition (MET) to reverse their EMT characteristics and regain its epithelial phenotype before they can colonize and form metastatic lesions [36–39]. However, it is also possible that the tumor cells at the metastatic sites were derived from tumor cells that have never undergone EMT [40]. Ligorio et al. recently demonstrated that CAFs could drive cancer cells towards more EMT and proliferative phenotypes. They further described that patient tumors with medium level (as oppose to low and high levels) of stromal content had the highest number of tumor glands with EMT characteristics [41]. In our study, the two primary tumors with the highest numbers of EMT cells did not have a high number of CAFs either, which is somewhat consistent with Ligorio and colleagues’ findings.

In agreement with the notion that EMT^+^ tumor cells tend to be more aggressive and resistant to chemotherapies, we found that patients with a high number of EMT^+^ cells have poorer outcomes (Fig. 5a). This is consistent with previous studies using bulk tissue transcriptomics that found PDAC subtypes with high mesenchymal gene expression signals (i.e., QM, squamous, or basal subtypes) have worse outcomes than the rest of the subtypes [30–32]. We also found that PDAC tumors have relatively low numbers of endothelial cells (Table 1). However, if a patient’s tumor is enriched for gene expression signals from endothelial cells, that patient would have a better survival than those who have low endothelial signaling (Fig. 5c). This finding supports the hypothesis that improved vascularity in PDAC can lead to better patient outcomes possibly due to better infiltration of immune cells and/or delivery of therapeutics [42]. Furthermore, we discovered that level of gene expression signals of all CAFs did not significantly correlate with patient survival (Fig. 5d). However, the gene expression signal of activated CAFs was significantly associated with patient survival (Fig. 5e), whereas the gene expression signal of the quiescent CAFs seems to be associated with relatively better patient survival (although it is not statistically significant) (Fig. 5f).

The three CAF subtypes (myCAF, iCAF, and apCAF) described by Elyada and colleagues provided important insights into the function of pancreatic CAFs [24, 25]. In our study, we identified 3 major CAF clusters: cluster 0 was enriched for markers for myCAF, but the two other clusters do not seem to enrich for markers for either iCAF or apCAF. We did identify cells that express some of the prominent markers for iCAF (e.g., IL6 and CXCL12) and apCAF (e.g., CD74 and HLA-DQA1), albeit they were scattered across all the 3 clusters (Fig. S19). The result for the apCAF is consistent with what was described by Elyada et al. in which the apCAFs formed a separate cluster among CAFs derived from murine PDAC, whereas apCAFs detected in human PDAC were scattered within the iCAF and myCAF clusters. Therefore, our study still supports the existent of 3 functional subtypes of CAFs in PDAC tumors, but the expression signatures of iCAFs and apCAFs are not distinct enough to drive the formation of separate clusters. One possible explanation for this difference between our study and those of Elyada et al. [24] and Ohlund et al. [25] is the different methods used for single-cell preparation before RNA sequencing. In our study, tumor tissues were dissociated by enzymatic digestion and mechanical force and then directly used for scRNA-Seq, whereas in the studies by Elyada and colleagues, cells were further processed after dissociation to enrich for CAFs by either flow cytometry or in vitro culture before scRNA-Seq.

## Conclusions

In summary, our work identifies significant inter- and intra-tumor heterogeneities in cellular compositions of PDAC tumors and between primary tumors and metastatic lesions. We also found that the cellular composition was an important factor in defining PDAC subtypes and significantly correlated with patient outcome. These findings provide valuable insights on PDAC microenvironment and could potentially inform the management of PDAC patients. Our study also suggests that single-cell transcriptome analysis can offer important clinical insights on cell subpopulations to develop novel therapeutic strategies for both targeted and immunotherapies.

## Supplementary information

**Additional file 1 **This file contains Supplementary Table S1 and Supplementary Figure S1 to S19. **Table S1:** Clinical histopathological parameters of patients. **Fig. S1:** Cell types identified using the SuperCT tool. **Fig. S2:** Analysis of cells in active cell cycle phases (S and G2/M phase). **Fig. S3:** Expression of epithelial cell marker (KRT19) and mesenchymal cell markers (CDH2, SNAI2, ZEB1, VIM, and FN1) in different cell clusters identified in the primary tumors. **Fig. S4:** Inferred copy number analysis of different cell types. **Fig. S5**: Distribution of fraction of mitochondrial genes in individual cells across different cell types. **Fig. S6:** Expression of cancer stem cell marker PROM1 (also known as CD133) in the cell clusters identified in primary tumors. **Fig. S7:** Ingenuity pathway analysis of signature genes unique to CAF (A) and EMT (B) cells. **Fig. S8:** Violin plots showing the expression of pancreatic epithelial (KRT19) and mesenchymal (CDH2, SNAI2, ZEB1, VIM, and FN1) marker genes in individual patients’ tumors. **Fig. S9:** Cell types identified in metastatic lesions by SuperCT. **Fig. S10:** Unsupervised clustering of cells from both primary and metastatic tumor tissues. **Fig. S11:** Violin plots show the expression patterns of the smooth muscle gene markers (RGS5, NOTCH3 and CSRP2) among the CAF clusters. **Fig. S12:** Characterization of tumor infiltrating lymphocytes (TILs) in the PDAC primary tumors. **Fig. S13:** Violin plots showing the expression of the Immunogenic subtype signature genes in different cell types identified in primary tumors. **Fig. S14:** SuperCT analysis revealed that the gene signatures that define the Exocrine subtype described in the Collisson study and the ADEX subtype described in the Bailey study are enriched in the acinar cells. **Fig. S15:** Violin plots showing the expression patterns of the classic subtype signature genes described in the Collisson study, progenitor subtype and squamous subtype signature genes described in the Bailey study across the primary tumors. **Fig. S16:** Violin plots showing the expression patterns of PDAC subtype specific gene signatures across the primary tumors for the QM subtype and Immunogenic subtype as described in the Bailey study. **Fig. S17:** Unsupervised clustering analysis of the scRNA-seq data using the signature gene sets that were reported to classify PDAC molecular subtypes.

**Additional file 2.** This file contains Supplementary Table S2 which lists the top 20 signature genes for each cell type identified from scRNA-seq.

**Additional file 3.** This file contains Supplementary Table S3 which lists the unique signature genes that define the CAF and EMT cell populations.

## Data Availability

The new datasets generated and analyzed during the current study have been deposited to the GEO database (Accession # GSE154778) [43]. The public datasets on bulk RNA-Seq analysis of PDAC patients were downloaded from the International Cancer Genome Consortium (ICGC) data portal [44]. The Australian cohort (PACA-AU) can be found at https://dcc.icgc.org/releases/release_20/Projects/PACA-AU. The Canadian cohort (PACA-CA) can be found at https://dcc.icgc.org/releases/release_20/Projects/PACA-CA. The US TCGA cohort (PAAD-US) can be found at https://dcc.icgc.org/releases/release_20/Projects/PAAD-US. The dataset from Peng et al. [13] was downloaded from Genome Sequence Archive (accession number: CRA001160) at https://bigd.big.ac.cn/bioproject/browse/PRJCA001063. The SuperCT cell type classifier [15] can be downloaded at https://github.com/weilin-genomics/SuperCT. and https://github.com/weilin-genomics/ rSuperCT. The Seruat R Package can be found at https://satijalab.org/seurat/.

## Abbreviations

ADEX: Aberrantly differentiated endocrine exocrine
CAF: Cancer-associated fibroblast
DC: Dendritic cell
ECM: Extracellular matrix
EMT: Epithelial to mesenchymal transition
ETC: Epithelial tumor cell
FACS: Fluorescence-activated cell sorting
ICGC: International Cancer Genome Consortium
IHC: Immunohistochemistry
MET: Metastasis
PBS: Phosphate buffer saline
PDAC: Pancreatic ductal adenocarcinoma
QM: Quasi-mesenchymal
scRNA-Seq: Single-cell RNA sequencing
TAM: Tumor-associated macrophage
TCGA: The Cancer Genome Atlas
TIL: Tumor-infiltrating lymphocyte
TME: Tumor microenvironment
tSNE: t-Distributed stochastic neighbor embedding
UMAP: Uniform Manifold Approximation and Projection
UMI: Unique molecular identifier

## References

[1] Pattabiraman DR, Weinberg RA (2014). Tackling the cancer stem cells - what challenges do they pose?. Nat Rev Drug Discov.

[2] Whatcott C, Han H, Posner RG, Von Hoff DD (2013). Tumor-stromal interactions in pancreatic cancer. Crit Rev Oncog.

[3] Doherty GJ, Tempero M, Corrie PG (2018). HALO-109-301: a phase III trial of PEGPH20 (with gemcitabine and nab-paclitaxel) in hyaluronic acid-high stage IV pancreatic cancer. Future Oncol.

[4] Hingorani SR, Zheng L, Bullock AJ, Seery TE, Harris WP, Sigal DS (2018). HALO 202: randomized phase II study of PEGPH20 plus nab-paclitaxel/gemcitabine versus nab-paclitaxel/gemcitabine in patients with untreated, metastatic pancreatic ductal adenocarcinoma. J Clin Oncol.

[5] Pure E, Lo A (2016). Can targeting stroma pave the way to enhanced antitumor immunity and immunotherapy of solid tumors?. Cancer Immunol Res.

[6] Hah N, Sherman MH, Yu RT, Downes M, Evans RM (2015). Targeting transcriptional and epigenetic reprogramming in stromal cells in fibrosis and cancer. Cold Spring Harb Symp Quant Biol.

[7] Bandura DR, Baranov VI, Ornatsky OI, Antonov A, Kinach R, Lou X (2009). Mass cytometry: technique for real time single cell multitarget immunoassay based on inductively coupled plasma time-of-flight mass spectrometry. Anal Chem.

[8] Bendall SC, Simonds EF, Qiu P, Amir el AD, Krutzik PO, Finck R (2011). Single-cell mass cytometry of differential immune and drug responses across a human hematopoietic continuum. Science..

[9] Spitzer MH, Nolan GP (2016). Mass cytometry: single cells, many features. Cell..

[10] Zheng GX, Terry JM, Belgrader P, Ryvkin P, Bent ZW, Wilson R (2017). Massively parallel digital transcriptional profiling of single cells. Nat Commun.

[11] Butler A, Hoffman P, Smibert P, Papalexi E, Satija R (2018). Integrating single-cell transcriptomic data across different conditions, technologies, and species. Nat Biotechnol.

[12] Stuart T, Butler A, Hoffman P, Hafemeister C, Papalexi E, Mauck WM (2019). Comprehensive integration of single-cell data. Cell.

[13] Peng J, Sun BF, Chen CY, Zhou JY, Chen YS, Chen H (2019). Single-cell RNA-seq highlights intra-tumoral heterogeneity and malignant progression in pancreatic ductal adenocarcinoma. Cell Res.

[14] Yu X, Chen YA, Conejo-Garcia JR, Chung CH, Wang X (2019). Estimation of immune cell content in tumor using single-cell RNA-seq reference data. BMC Cancer.

[15] Xie P, Gao M, Wang C, Zhang J, Noel P, Yang C (2019). SuperCT: a supervised-learning framework for enhanced characterization of single-cell transcriptomic profiles. Nucleic Acids Res.

[16] Patel AP, Tirosh I, Trombetta JJ, Shalek AK, Gillespie SM, Wakimoto H (2014). Single-cell RNA-seq highlights intratumoral heterogeneity in primary glioblastoma. Science..

[17] Yu G, Wang LG, Han Y, He QY (2012). clusterProfiler: an R package for comparing biological themes among gene clusters. OMICS..

[18] Weinstein JN, Collisson EA, Mills GB, Shaw KR, Ozenberger BA, Cancer Genome Atlas Research N (2013). The Cancer Genome Atlas Pan-Cancer analysis project. Nat Genet.

[19] Hudson TJ, Anderson W, Artez A, Barker AD, Bell C, International Cancer Genome C (2010). International network of cancer genome projects. Nature..

[20] Li H, van der Leun AM, Yofe I, Lubling Y, Gelbard-Solodkin D, van Akkooi ACJ (2019). Dysfunctional CD8 T cells form a proliferative, dynamically regulated compartment within human melanoma. Cell.

[21] Becht E, McInnes L, Healy J, Dutertre CA, Kwok IWH, Ng LG, et al. Dimensionality reduction for visualizing single-cell data using UMAP. Nat Biotechnol. 2019;37(1):38–44. 10.1038/nbt.4314 30531897

[22] Tirosh I, Izar B, Prakadan SM, Wadsworth MH, Treacy D, Trombetta JJ (2016). Dissecting the multicellular ecosystem of metastatic melanoma by single-cell RNA-seq. Science..

[23] Karaayvaz M, Cristea S, Gillespie SM, Patel AP, Mylvaganam R, Luo CC (2018). Unravelling subclonal heterogeneity and aggressive disease states in TNBC through single-cell RNA-seq. Nat Commun.

[24] Elyada E, Bolisetty M, Laise P, Flynn WF, Courtois ET, Burkhart RA (2019). Cross-species single-cell analysis of pancreatic ductal adenocarcinoma reveals antigen-presenting cancer-associated fibroblasts. Cancer Discov.

[25] Ohlund D, Handly-Santana A, Biffi G, Elyada E, Almeida AS, Ponz-Sarvise M (2017). Distinct populations of inflammatory fibroblasts and myofibroblasts in pancreatic cancer. J Exp Med.

[26] Raza A, Franklin MJ, Dudek AZ (2010). Pericytes and vessel maturation during tumor angiogenesis and metastasis. Am J Hematol.

[27] Brown JM, Recht L, Strober S (2017). The promise of targeting macrophages in cancer therapy. Clin Cancer Res.

[28] Figueiredo CR, Azevedo RA, Mousdell S, Resende-Lara PT, Ireland L, Santos A (2018). Blockade of MIF-CD74 signalling on macrophages and dendritic cells restores the antitumour immune response against metastatic melanoma. Front Immunol.

[29] Lankadasari MB, Mukhopadhyay P, Mohammed S, Harikumar KB (2019). TAMing pancreatic cancer: combat with a double edged sword. Mol Cancer.

[30] Collisson EA, Sadanandam A, Olson P, Gibb WJ, Truitt M, Gu S (2011). Subtypes of pancreatic ductal adenocarcinoma and their differing responses to therapy. Nat Med.

[31] Bailey P, Chang DK, Nones K, Johns AL, Patch AM, Gingras MC (2016). Genomic analyses identify molecular subtypes of pancreatic cancer. Nature..

[32] Moffitt RA, Marayati R, Flate EL, Volmar KE, Loeza SG, Hoadley KA (2015). Virtual microdissection identifies distinct tumor- and stroma-specific subtypes of pancreatic ductal adenocarcinoma. Nat Genet.

[33] Gaianigo N, Melisi D, Carbone C. EMT treatment resistance in pancreatic cancer. Cancers. 2017;9(9):122. 10.3390/cancers9090122 PMC5615337 28895920

[34] Wang Z, Li Y, Ahmad A, Banerjee S, Azmi AS, Kong D (2011). Pancreatic cancer: understanding and overcoming chemoresistance. Nat Rev Gastroenterol Hepatol.

[35] Hosein AN, Huang H, Wang Z, Parmar K, Du W, Huang J, et al. Cellular heterogeneity during mouse pancreatic ductal adenocarcinoma progression at single-cell resolution. JCI Insight. 2019;5(16):e129212. 10.1172/jci.insight.129212 PMC6777805 31335328

[36] Steeg PS (2016). Targeting metastasis. Nat Rev Cancer.

[37] Yao D, Dai C, Peng S (2011). Mechanism of the mesenchymal-epithelial transition and its relationship with metastatic tumor formation. Mol Cancer Res.

[38] Ling L, Chen L, Zhang C, Gui S, Zhao H, Li Z (2018). High glucose induces podocyte epithelial-to-mesenchymal transition by demethylation-mediated enhancement of MMP9 expression. Mol Med Rep.

[39] Lu W, Kang Y (2019). Epithelial-mesenchymal plasticity in cancer progression and metastasis. Dev Cell.

[40] Zheng X, Carstens JL, Kim J, Scheible M, Kaye J, Sugimoto H (2015). Epithelial-to-mesenchymal transition is dispensable for metastasis but induces chemoresistance in pancreatic cancer. Nature..

[41] Ligorio M, Sil S, Malagon-Lopez J, Nieman LT, Misale S, Di Pilato M (2019). Stromal microenvironment shapes the intratumoral architecture of pancreatic cancer. Cell.

[42] Katsuta E, Qi Q, Peng X, Hochwald SN, Yan L, Takabe K (2019). Pancreatic adenocarcinomas with mature blood vessels have better overall survival. Sci Rep.

[43] Lin W, Noel P, Han H. Single-cell transcriptomics analysis of pancreatic primary tumor and metastatic biopsy tissues. Gene Expression Omnibus (GEO). 2020;Accession # GSE154778.

[44] Zhang J, Bajari R, Andric D, Gerthoffert F, Lepsa A, Nahal-Bose H (2019). The international cancer genome consortium data portal. Nat Biotechnol.

