# Supplementary material for: Single-cell transcriptome analysis of tumor and stromal compartments of pancreatic ductal adenocarcinoma primary tumors and metastatic lesions
Source: Genome Med. 2020 Sep 29;12:80. doi: 10.1186/s13073-020-00776-9 (PMC7523332; doi:10.1186/s13073-020-00776-9)
Supplement: Supplementary file 1 — Additional file 1 This file contains Supplementary Table S1 and Supplementary Figure S1 to S19. Table S1: Clinical histopathological parameters of patients. Fig. S1: Cell types identified using the SuperCT tool. Fig. S2: Analysis of cells in active cell cycle phases (S and G2/M phase). Fig. S3: Expression of epithelial cell marker (KRT19) and mesenchymal cell markers (CDH2, SNAI2, ZEB1, VIM, and FN1) in different cell clusters identified in the primary tumors. Fig. S4: Inferred copy number analysis of different cell types. Fig. S5: Distribution of fraction of mitochondrial genes in individual cells across different cell types. Fig. S6: Expression of cancer stem cell marker PROM1 (also known as CD133) in the cell clusters identified in primary tumors. Fig. S7: Ingenuity pathway analysis of signature genes unique to CAF (A) and EMT (B) cells. Fig. S8: Violin plots showing the expression of pancreatic epithelial (KRT19) and mesenchymal (CDH2, SNAI2, ZEB1, VIM, and FN1) marker genes in individual patients’ tumors. Fig. S9: Cell types identified in metastatic lesions by SuperCT. Fig. S10: Unsupervised clustering of cells from both primary and metastatic tumor tissues. Fig. S11: Violin plots show the expression patterns of the smooth muscle gene markers (RGS5, NOTCH3 and CSRP2) among the CAF clusters. Fig. S12: Characterization of tumor infiltrating lymphocytes (TILs) in the PDAC primary tumors. Fig. S13: Violin plots showing the expression of the Immunogenic subtype signature genes in different cell types identified in primary tumors. Fig. S14: SuperCT analysis revealed that the gene signatures that define the Exocrine subtype described in the Collisson study and the ADEX subtype described in the Bailey study are enriched in the acinar cells. Fig. S15: Violin plots showing the expression patterns of the classic subtype signature genes described in the Collisson study, progenitor subtype and squamous subtype signature genes described in the Bailey study across the primary [file 13073_2020_776_MOESM1_ESM.docx]

**Supplementary TAble**

**
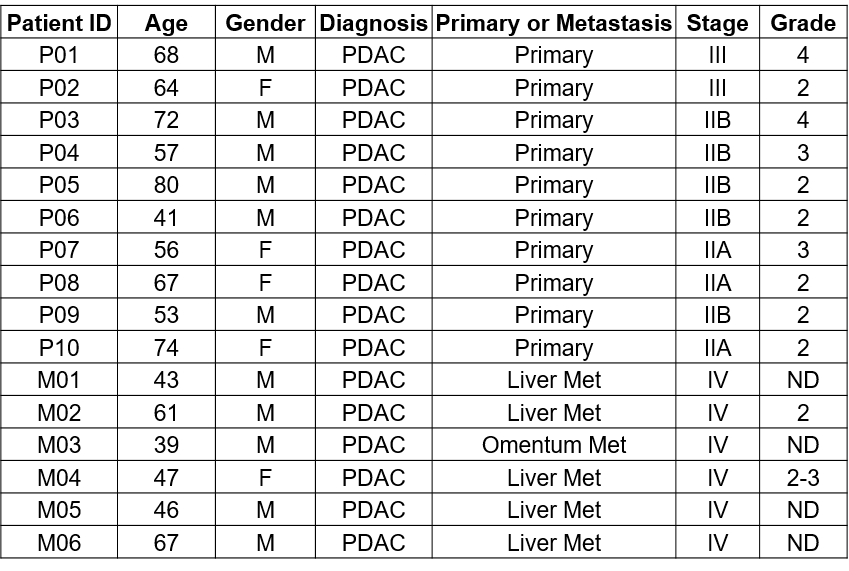
**

Met: metastasis; ND: Not determined

**Table S1: Clinical histopathological parameters of patients**

**Supplementary Figures**

**
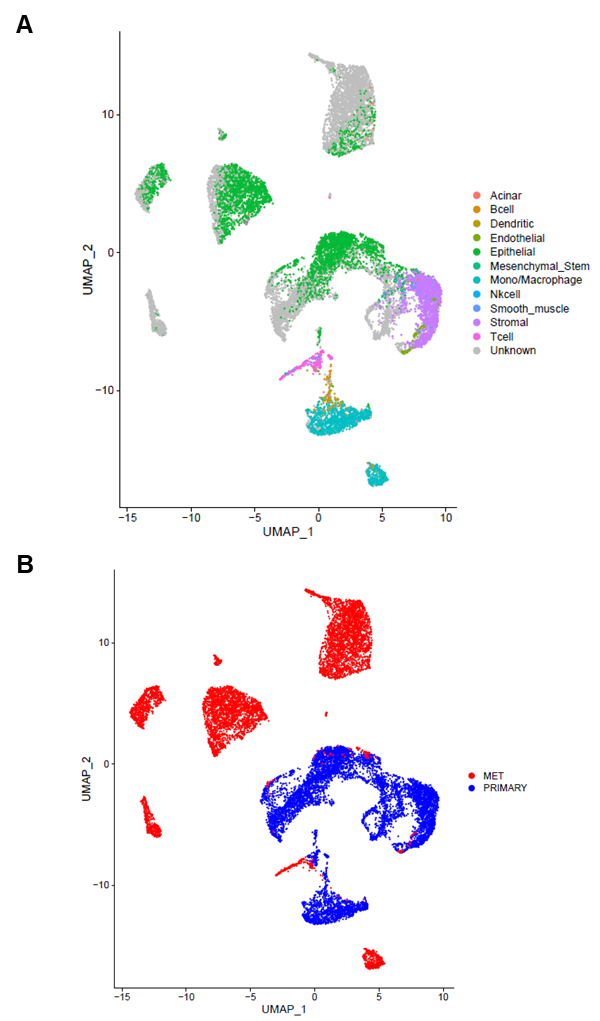
**

**Fig. S1.** Cell type assignment using the SuperCT tool. A) Cell types predicted by SuperCT. B) Clustering of cells from primary tumors and metastatic biopsies.


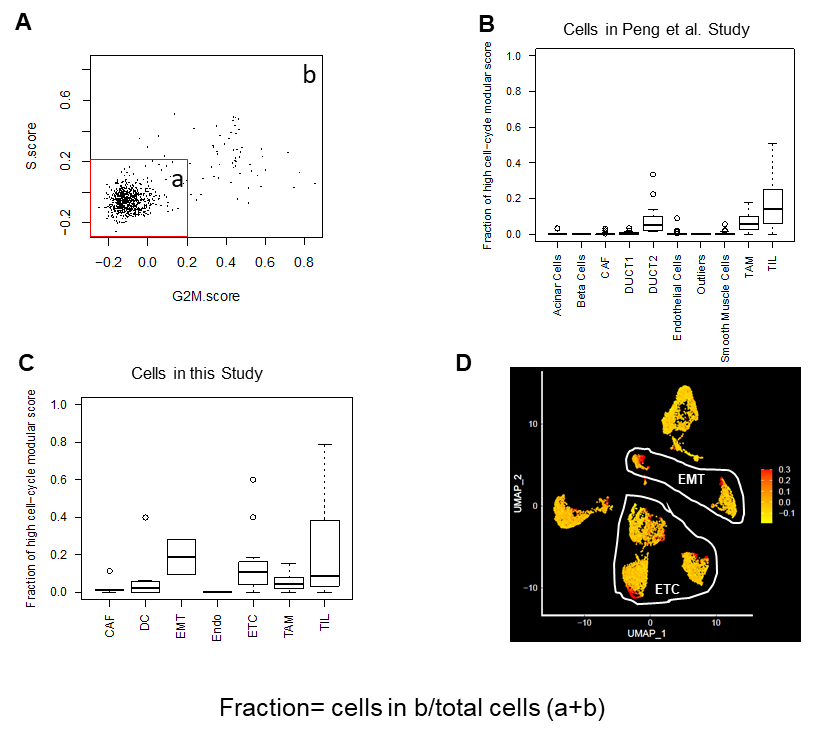


**Fig. S2.** Analysis of cells in active cell cycle phases (S and G2/M phase). A) Cells that have high scores in either G2/M or S phase specific gene signature expression were gated and counted based on single cell RNA sequencing data. The fraction of cells that were in either G2/M or S phase for each cell type was then plotted in a box plot (B and C). B) the fraction of cells with high cell-cycle modular scores in different cell types identified in the study by Peng et al (Reference #16). C) The fraction of cells with high cell-cycle modular scores in different cell types within the primary tumors in this study. D) UMAP plot of cell-cycle modular scores as intensity colors for the cells identified in the primary tumors (red color indicates a high modular score).

·


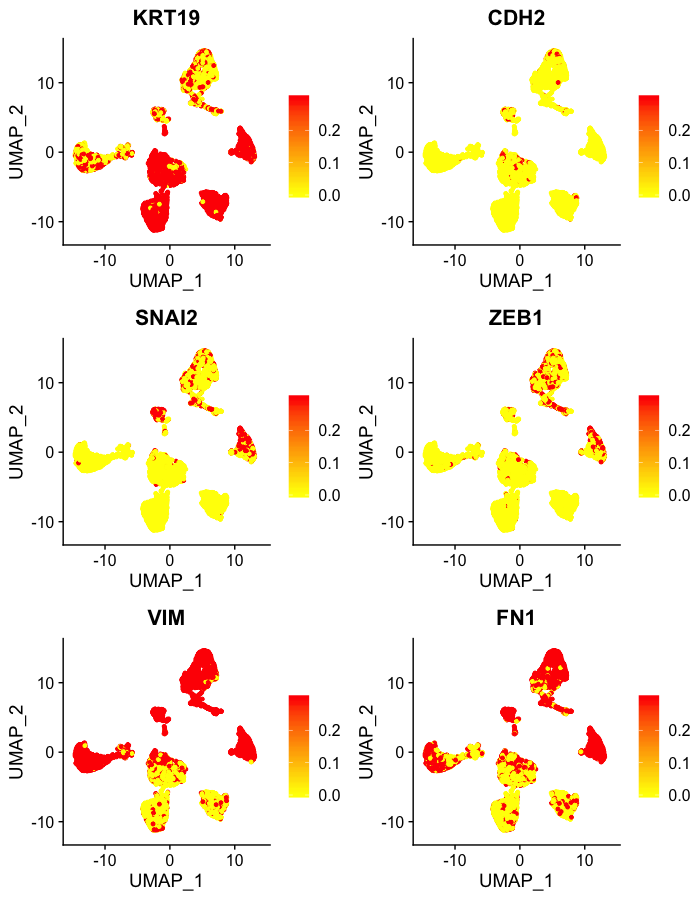


**Fig. S3.** Expression of epithelial cell marker (KRT19) and mesenchymal cell markers (CDH2, SNAI2, ZEB1, VIM, and FN1) in different cell clusters identified in the primary tumors. The feature expression values were calculated based on the ‘logNormalize’ method and rescaled using generalized linear model based on negative binomial distribution.

**
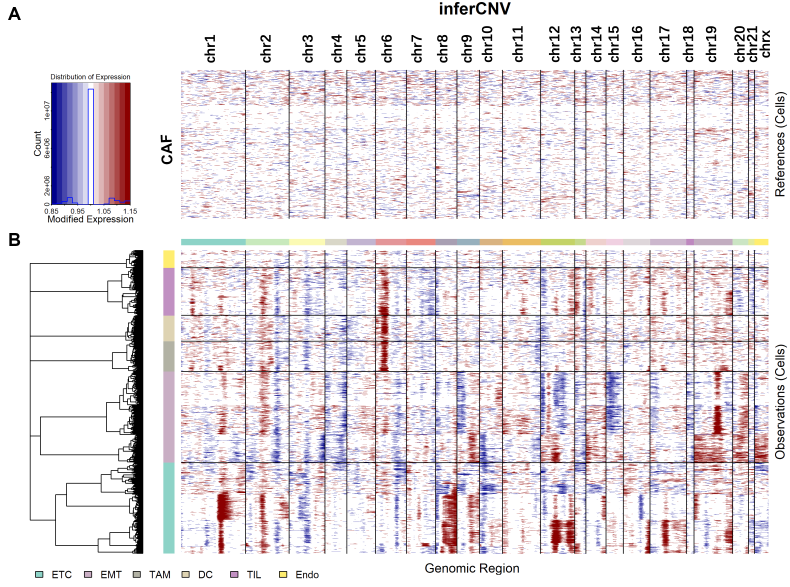
**

**Fig. S4.** Inferred copy number analysis of different cell types. A) Cancer associated fibroblasts (CAFs) demonstrated a uniform inferred copy numbers across the cells from different patients, therefore CAFs were selected to serve as a reference for the copy number comparison. B) Different cell types (as indicated by the color bar on the left) were compared to CAFs on the inferred copy number variations. The two cancer cell subsets (ETC and EMT) showed substantial CNVs on multiple chromosomes. Interestingly the immune cell subsets showed significant inferred copy number gains on Chromosome 6, which is consistent with the fact that many immune related genes such as the major histocompatibility complex (MHC) genes are located on Chromosome 6. These genes are known to be highly expressed in immune cells.


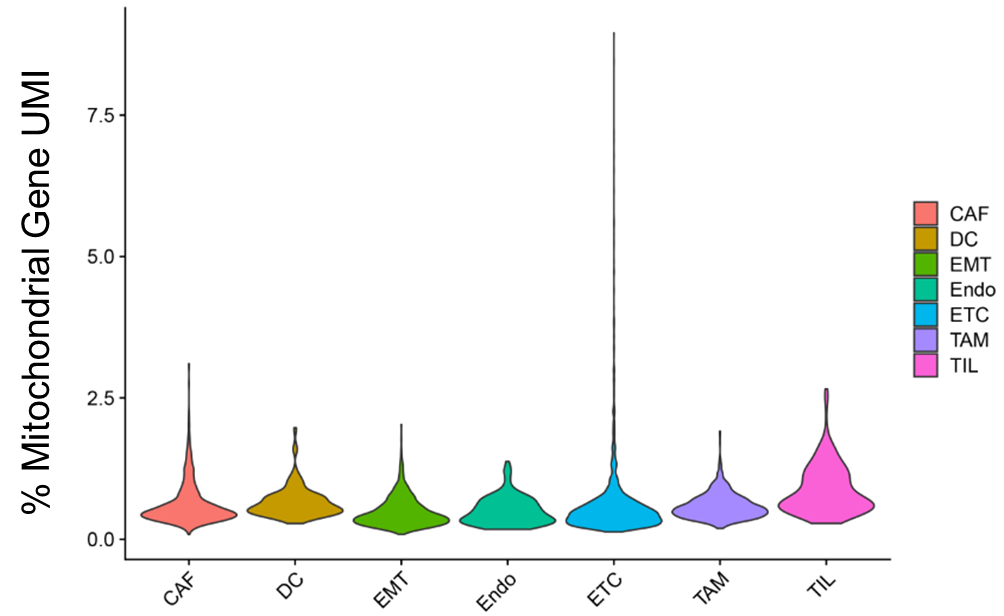


**Fig. S5.** Distribution of fraction of mitochondrial genes in individual cells across different cell types. % Mitochondrial Gene UMI = # of mitochondrial gene UMIs / # of total UMIs detected in each cell.


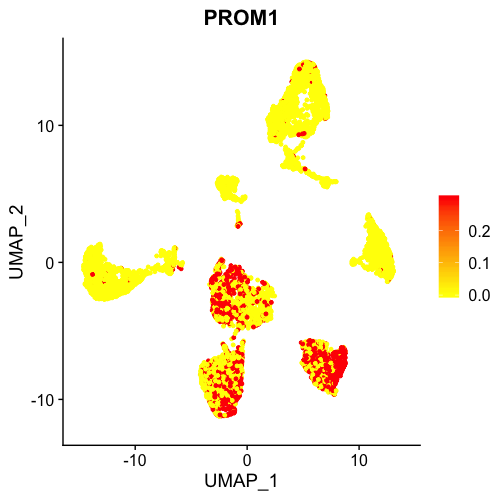


**Fig. S6.** Expression of cancer stem cell marker PROM1 (also known as CD133) in the cell clusters identified in primary tumors.


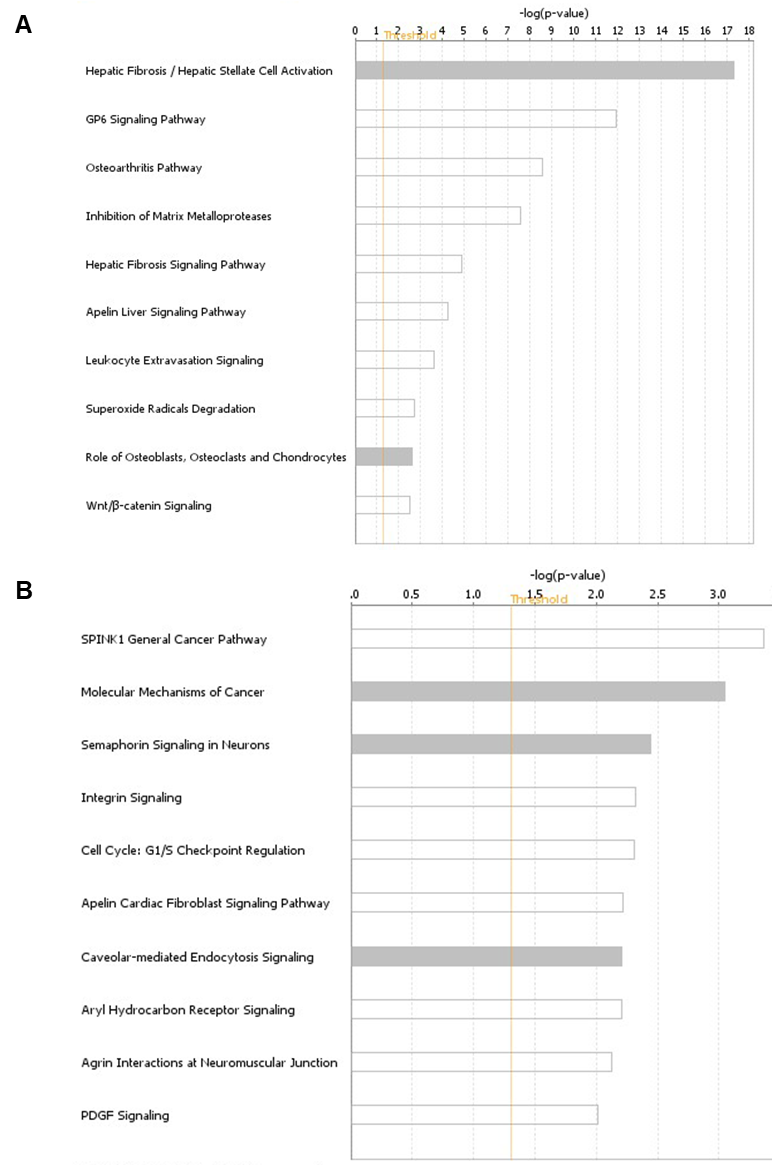


**Fig. S7.** Ingenuity pathway analysis of signature genes unique to CAF (A) and EMT (B) cells. The genes included in the analysis are listed in Table S3. Gray bars indicates no activity pattern available.


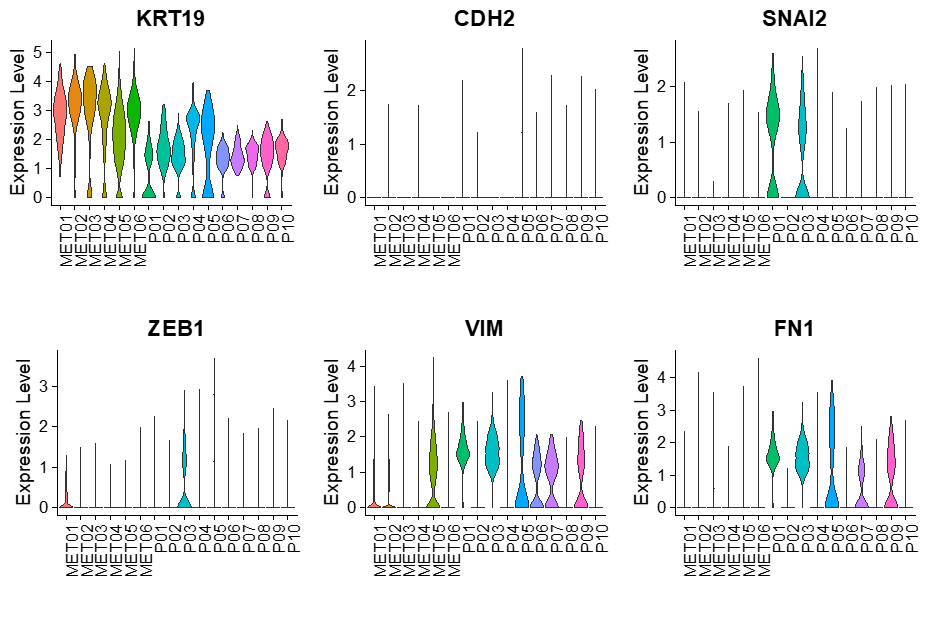


**Fig. S8.** Violin plots showing the expression of pancreatic epithelial (KRT19) and mesenchymal (CDH2, SNAI2, ZEB1, VIM, and FN1) marker genes in individual patients’ tumors.


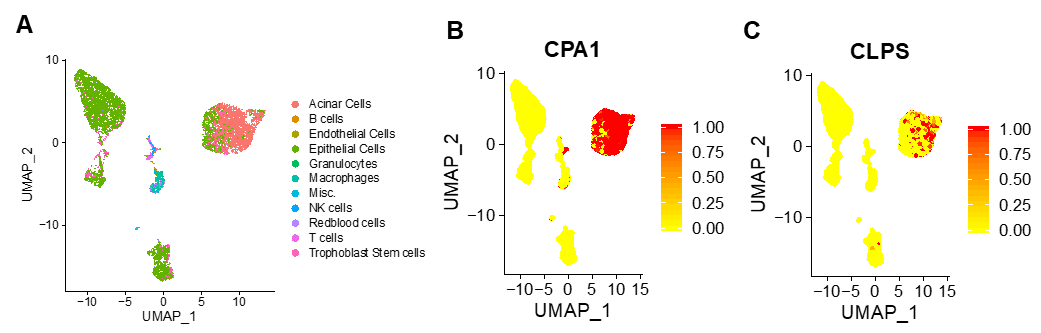


**Fig. S9.** Cell types identified in metastatic lesions by SuperCT. Besides epithelial cells, acinar cells are one of the larger groups of cells identified (A). The acinar cells express high levels of CPA1 and CLPS genes (B and C).

**Fig. S10.** Unsupervised clustering of cells from both primary and metastatic tumor tissues. The UMAP plot shows the clusters with cells color-coded based on their assigned cell type. The clustering of different cell types is largely maintained and the epithelial tumor cells from some of the patients still form clusters based on their patient of origin (blue-colored cells).


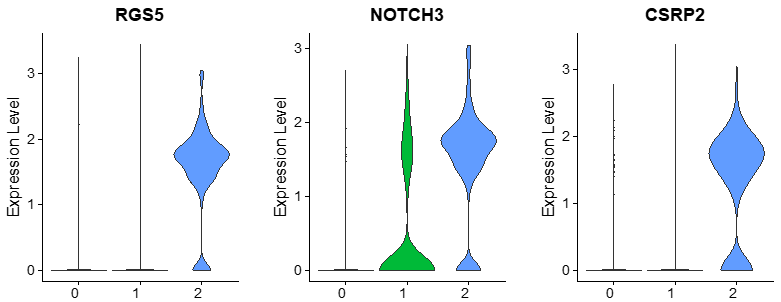


**Fig. S11.** Violin plots show the expression patterns of the smooth muscle gene markers (RGS5, NOTCH3 and CSRP2) among the CAF clusters.


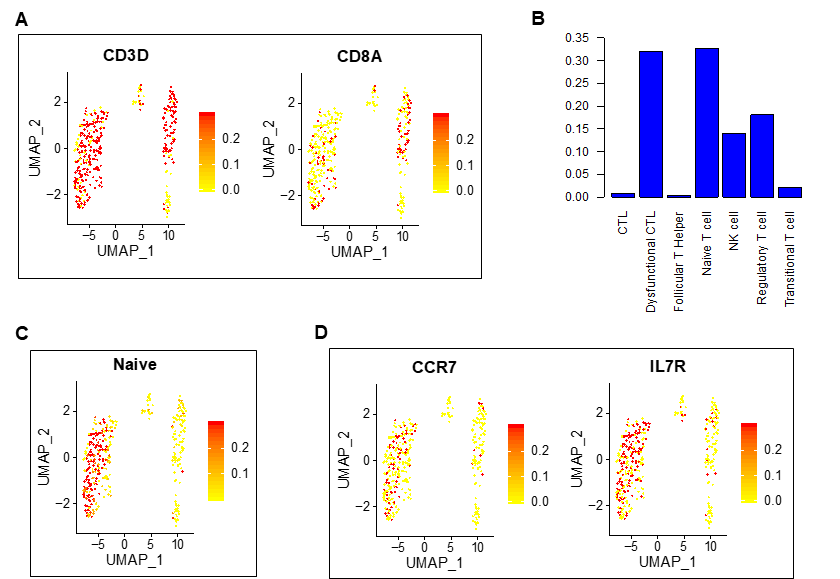


**Fig. S12.** Characterization of tumor infiltrating lymphocytes (TILs) in the PDAC primary tumors. A) TILs are mostly CD8-. B) Fractions of different subtypes of TILs identified in the primary tumors using SuperCT. The fraction was calculated by dividing the number of cells for a given subtype from the total number of TILs identified. CTL: cytotoxic T cells. NK: Natural killer T cells. C) Cluster 1 (Figure 3B) is enriched with the naïve T cells. D) The expression of Naïve T cell markers (CCR7 and IL7R) is most in cells in Cluster 1.


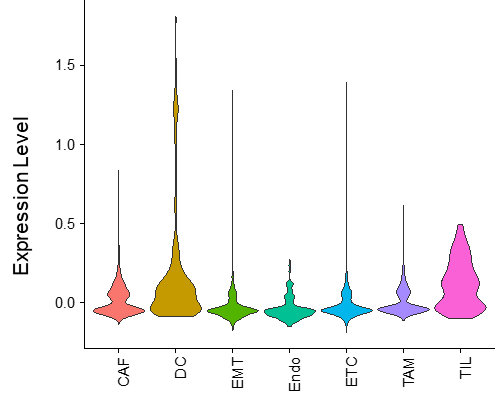


**Fig. S13.** Violin plots showing the expression of the Immunogenic subtype signature genes in different cell types identified in primary tumors. Dendritic cells (DC) and tumor infiltrating lymphocytes (TIL) have the highest expression.


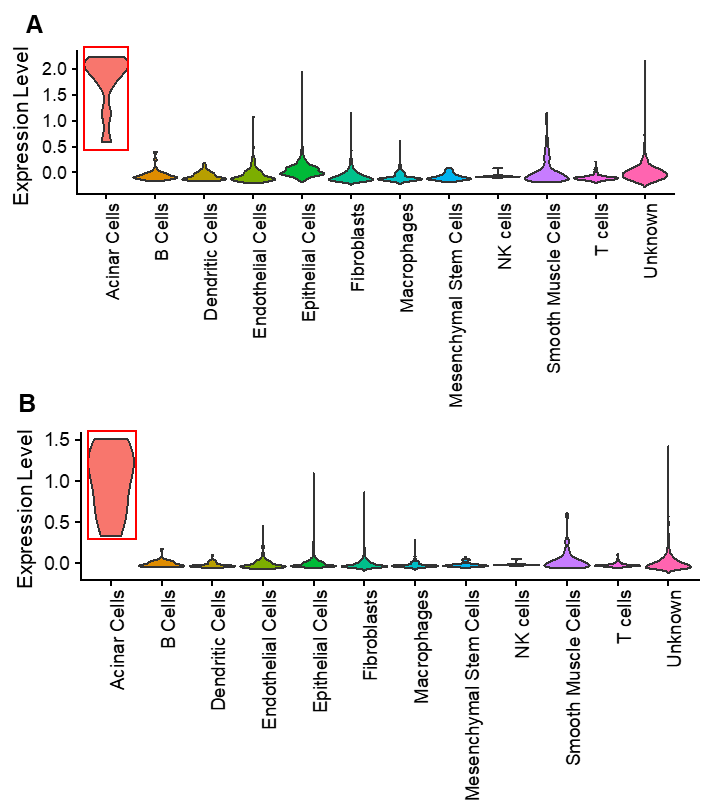


**Fig. S14.** SuperCT analysis revealed that the gene signatures that define the Exocrine subtype described in the Collisson study (A) and the ADEX subtype described in the Bailey study (B) are enriched in the acinar cells.

**
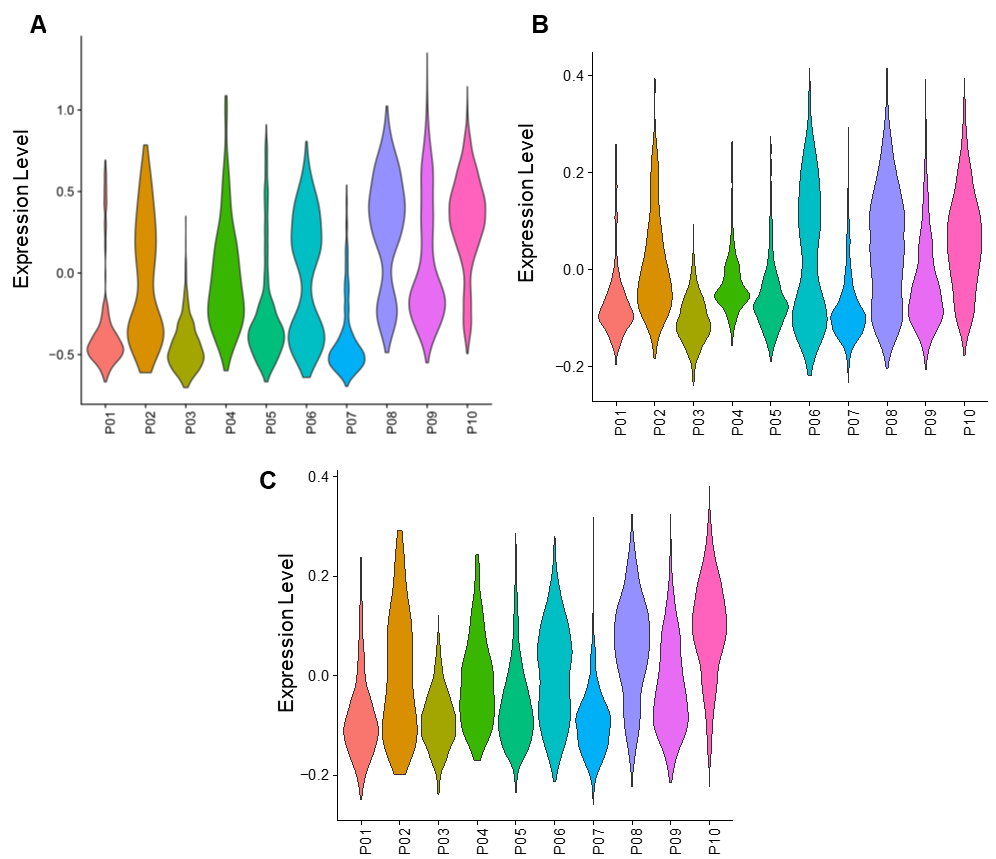
**

**Fig. S15.** Violin plots showing the expression patterns of the Classic subtype (A) signature genes described in the Collisson study, Progenitor subtype (B) and Squamous subtype (C) signature genes described in the Bailey study across the primary tumors.


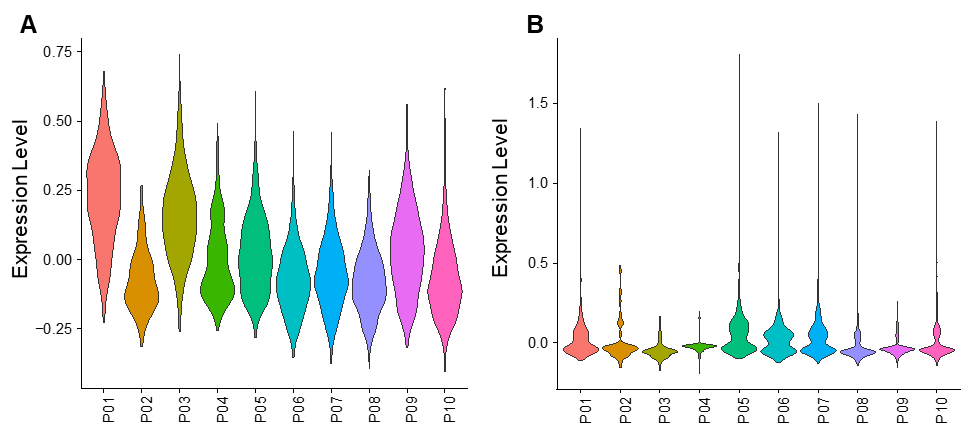


**Fig. S16.** Violin plots showing the expression patterns of PDAC subtype specific gene signatures across the primary tumors for the QM subtype (A) and Immunogenic subtype (B) as described in the Bailey study.


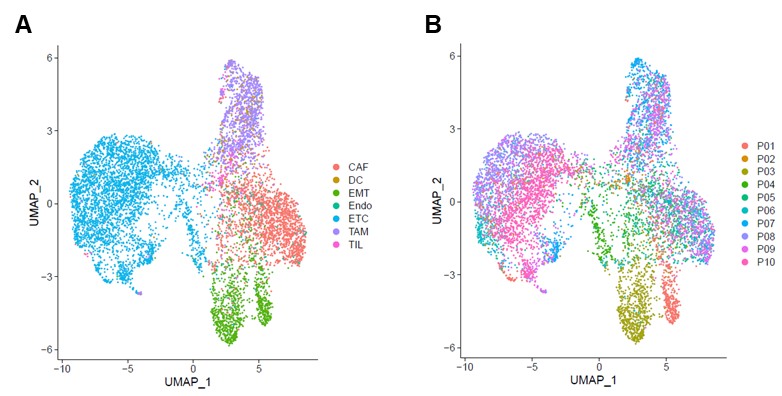


**Fig. S17.** Unsupervised clustering analysis of the scRNA-seq data using the signature gene sets that were reported to classify PDAC molecular subtypes. A) UMAP plot of the clustering analysis with cells color-coded by the cell types identified using the whole transcriptome. B) UMAP plot of the clustering analysis with cells color-coded by their patient of origin.


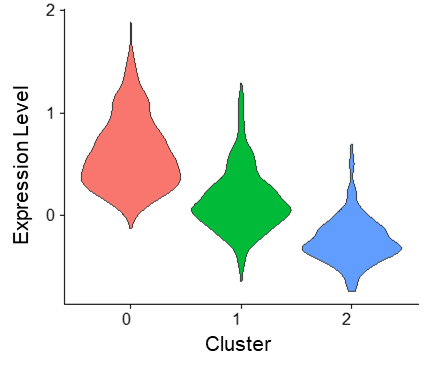


**Fig. S18.** Expression of the activated CAF gene signature in different sub-clusters of CAFs from primary tumors. Cluster 0 (Figure 2C) shows the highest expression level.


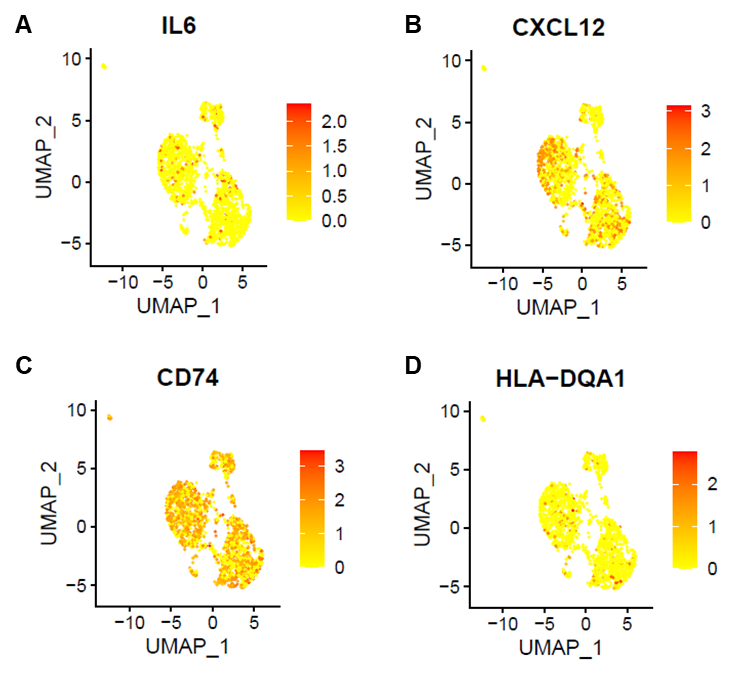


**Fig.** **S19**. Expression of iCAF markers, IL6 (A) and CXCL12 (B), and apCAF markers, CD74 (C) and HLA-DQA1 (D) across the different CAF clusters. Red color indicates high expression.
